# *BnPLP1* Positively Regulates Flowering Time, Plant Height, and Main Inflorescence Length in *Brassica napus*

**DOI:** 10.3390/genes14122206

**Published:** 2023-12-13

**Authors:** Ting Ding, Lei Cai, Yuqi He, Yuanhong Li, Entang Tian, Qianhui Zhou, Xufan Zhou, Xiaodong Wang, Kunjiang Yu, Xinjie Shen

**Affiliations:** 1 College of Agriculture, Guizhou University, Guiyang 550025, China; dingting1213@163.com (T.D.); caileixy@163.com (L.C.); yqhe1972@163.com (Y.H.); liyuanh31@163.com (Y.L.); erictian121@163.com (E.T.); qianhuizhou123@163.com (Q.Z.); xufanzhou123@163.com (X.Z.); 2 Center for Research and Development of Fine Chemical of Guizhou University, Guiyang 550025, China; 3 Institute of Industrial Crops, Jiangsu Academy of Agricultural Sciences, Key Laboratory of Cotton and Rapeseed, Ministry of Agriculture and Rural Affairs, Nanjing 210014, China; xdwang120@163.com

**Keywords:** *Brassica napus*, rapeseed, flowering time, plant height, main inflorescence length, protein prenylation

## Abstract

Protein prenylation mediated by the *Arabidopsis thaliana PLURIPETALA* (*AtPLP*) gene plays a crucial role in plant growth, development, and environmental response by adding a 15-carbon farnesyl group or one to two 20-carbon geranylgeranyl groups onto one to two cysteine residues at the C-terminus of the target protein. However, the homologous genes and their functions of *AtPLP* in rapeseed are unclear. In this study, bioinformatics analysis and gene cloning demonstrated the existence of two homologous genes of *AtPLP* in the *Brassica napus* L. genome, namely, *BnPLP1* and *BnPLP2*. Evolutionary analysis revealed that *BnPLP1* originated from the *B. rapa* L. genome, while *BnPLP2* originated from the *B. oleracea* L. genome. Genetic transformation analysis revealed that the overexpression of *BnPLP1* in *Arabidopsis* plants exhibited earlier flowering initiation, a prolonged flowering period, increased plant height, and longer main inflorescence length compared to the wild type. Contrarily, the downregulation of *BnPLP1* expression in *B. napus* plants led to delayed flowering initiation, shortened flowering period, decreased plant height, and reduced main inflorescence length compared to the wild type. These findings indicate that the *BnPLP1* gene positively regulates flowering time, plant height, and main inflorescence length. This provides a new gene for the genetic improvement of flowering time and plant architecture in rapeseed.

## 1. Introduction

*Brassica napus*, also known as rapeseed (AACC, 2n = 38), is one of the most important oilseed crops worldwide, providing people with healthy and delicious edible oils [1,2]. Flowering time, plant height, and main inflorescence length are three important traits closely associated with rapeseed yield [3,4,5,6]. Early flowering and shortening the flowering period can to some extent promote early maturity in winter rapeseed seeds, thereby avoiding a decrease in oil production caused by high temperatures during the seed filling period. Plant dwarfing can enhance the lodging resistance of rapeseed in the field, ensuring the growth quality of rapeseed, contributing to the improvement of rapeseed yield and quality, and facilitating mechanized harvesting [7]. Therefore, early maturity, long flowering periods, and dwarfing have become breeding goals pursued by more and more breeders in recent years [4,5]. Exploring more genes involved in regulating the flowering time and plant height of rapeseed can benefit breeders in creating new rapeseed varieties with different flowering times and/or plant heights through molecular breeding methods.

Flowering time is regulated by an intricate genetic network that integrates the response to diverse external and internal conditions [8]. Previous literature reviews have primarily outlined the flowering genetic network through the identification of six key regulatory pathways: vernalization, photoperiod, ambient temperature, autonomous, gibberellin (GA), and age-dependent pathways [8,9,10,11]. Many genes involved in these pathways have been identified in *Arabidopsis thaliana*. However, despite the numerous QTLs reported to be associated with flowering time in *B. napus*, only a few genes, such as *FLOWERING LOCUS T* [12], *FLOWERING LOCUS C* [13,14,15], *CONSTANS* [16], *FRIGIDA* [17,18], *BnNAC485* [19], and *CYCLING DOF FACTOR 1* [20], have been cloned. A recent review has summarized the genes involved in the regulation of flowering time in rapeseed, and many members of the MADS-box gene family have been included [21]. Recent studies have further revealed the effects of sequence variations in different members of the MADS-box gene family on flowering time in both *B. napus* [22] and *B. oleracea* L. [23,24]. In addition to MADS-box genes, the genetic improvement of flowering time in rapeseed still requires the identification of more genes.

Plant height is an essential component of plant architecture and plays a crucial role in increasing the yield of rapeseed [4,5]. The main inflorescence length of *Brassica napus* L. is an important component of plant height and is closely related to the yield of rapeseed [25]. A previous review indicated that the plant height of rapeseed is primarily associated with hormone synthesis and signal transduction within the body, while also being influenced by the environment [26]. Studies on *Arabidopsis* and rice have shown that gibberellins (GAs) and auxins (Auxs) are the two most important hormones affecting plant height [27,28], while brassinosteroids (BRs) and strigolactones (SLs) also play significant roles in the development of plant height morphology [29,30]. Recently, extensive research on the genetic study of plant height in *Brassica napus* has identified numerous quantitative trait loci (QTLs) on all 19 chromosomes [31,32,33,34,35,36,37,38,39,40,41,42,43,44]. However, only a few dwarfing genes involved in GA or IAA signal transduction pathways have been cloned, including *BnaA3.IAA7* [25,45], *BnaA06.RGA* [46], and *BnaC07.RGA*s [47]. In addition, the genetic analysis of the main inflorescence length indicated that this trait is mainly under genetic regulation, and is less influenced by the environment [42]. The identified QTLs related to the main inflorescence length are primarily distributed on chromosomes A02, A05, C01, and C06 [39,43].

The encoded products of the *PLURIPETALA* (*PLP*) gene are the protein farnesyltransferase (PFT) and protein geranylgeranyltransferase-I (PGGT-I) [48]. PFT and PGGT-I are two of the key enzymes involved in regulating protein prenylation in plant cells [49,50]. Genetic analysis in *Arabidopsis* has revealed the important roles of PFT and PGGT-I in plant growth, development, and environmental responses [51,52]. PFT and PGGT-I share a common α subunit (PLP protein), but have distinct β subunits that are distantly related in sequence (usually 25–35% similarity) and that determine substrate specificity [51,52]. In *Arabidopsis*, mutations in the *PLP* (known as *AtPLP*) gene result in abnormal plant growth and reproductive development, including slow plant growth, delayed flowering, flattened stems, shortened internode length, enlarged apical meristems, and increased numbers of floral organs [48]. Bioinformatic analysis indicates that *Arabidopsis* contains up to 700 proteins with the required structural domain for prenylation (at least one cysteine residue among the four amino acid residues at the C-terminal) [53]. Functional predictions suggest that these proteins play roles in various biological processes, including gene transcription regulation, hormone biosynthesis and signaling, protein folding, etc. [53]. However, only a small fraction of these proteins have had their functions experimentally confirmed [51,52]. In rapeseed, previous studies have shown that the downregulation of *PLP* (known as *BnPLP*) in shoots and roots can enhance drought resistance in plants [54]. However, the effects of *BnPLP* on other traits of rapeseed, particularly flowering time, plant height, and main inflorescence length, are still unknown.

In this study, two homologous genes of *AtPLP* were cloned from the DNA and cDNA of a semi-winter ecotype *B. napus* “APL01”, and they were named *BnPLP1* and *BnPLP2*, respectively. Furthermore, transgenic *Arabidopsis* plants overexpressing *BnPLP1* and RNA interference (RNAi) rapeseed plants with downregulated *BnPLP1* expression were generated to investigate the effects of *BnPLP1* on flowering time, plant height, and main inflorescence length in *B. napus*. This research outcome contributes to the elucidation of the function of the *BnPLP1* gene in *B. napus*. Additionally, it outlines potential applications of protein prenylation in the molecular breeding of *B. napus*, including early maturation and dwarfing breeding.

## 2. Results

### 2.1. Gene Cloning and Sequence Analysis of BnPLPs

**Figure 1:**
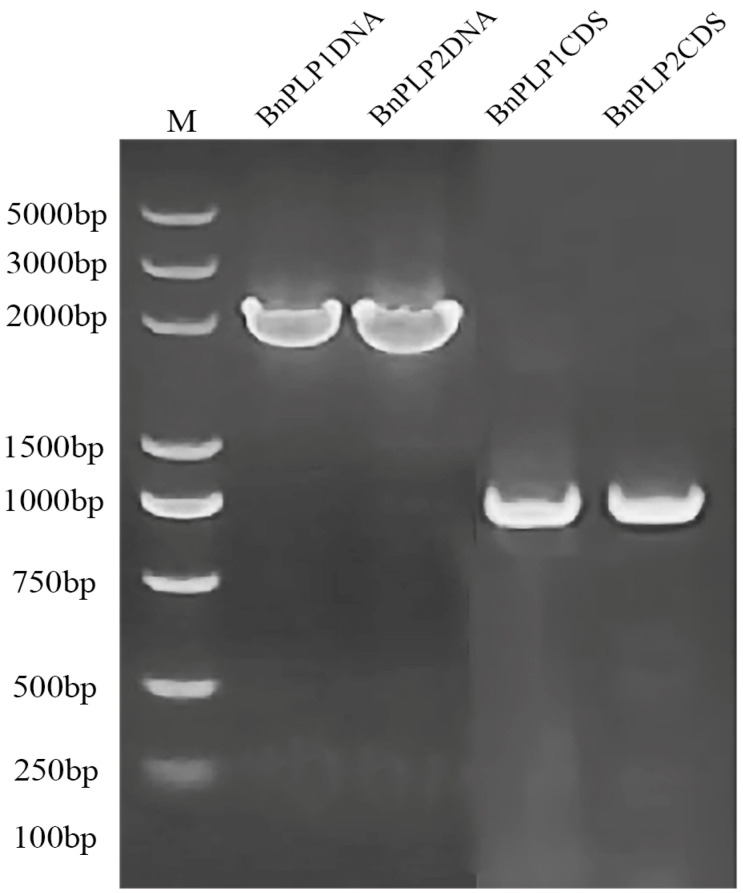
PCR cloning of the genomic DNA and coding sequences of the *BnPLP1* and *BnPLP2* genes.

Two homologous genes of *AtPLP* (AT3G59380), namely, BnaA01g18430D and BnaC01g41870D, in *B. napus* “Darmor”, and two homologous genes, namely, BnaA01T0195400ZS and BnaC01G0246300ZS, in “ZS11”, were retrieved by aligning the coding sequences (CDS) of *AtPLP* to the genomic databases of these two varieties. Based on the gene sequence information of *BnPLPs* in “Darmor” and “ZS11”, two pairs of primers, PR1 and PR2 (Table S1), were designed for cloning *BnPLPs* from the DNA of the rapeseed variety “APL01”. The results suggest that a 2145 bp fragment and a 2056 bp fragment were cloned from the DNA of “APL01”, with a sequence identity of 72.6% (Figure 1, Supplementary File S1). Additionally, another two pairs of primers, PR3 and PR4 (Table S1), were designed for cloning the full-length CDS of *BnPLPs*. The results show that two CDSs of *BnPLPs*, with lengths of 978 bp each, were cloned from the cDNA of “APL01”, and the sequence identity between them was 96.5% (Figure 1, Supplementary File S2). Subsequently, these two genes were named *BnPLP1* (corresponding to this DNA segment of 2145 bp) and *BnPLP2* (corresponding to this DNA segment of 2056 bp). Structural domain analysis revealed that both BnPLP1 and BnPLP2 proteins contain at least four protein prenyltransferase α subunit repeats within the range of the 55th to the 235th amino acid (Figure S1).

**Figure 2:**
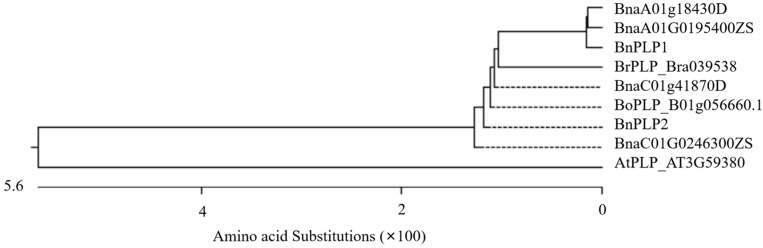
Evolutionary analysis of the *PLP* gene in *Arabidopsis thaliana*, *Brassica rapa*, *Brassica oleracea*, and *Brassica napus*. The genetic distance between genes is calculated using the neighbor-joining method, and an evolutionary tree is constructed based on the distance matrix. Branches on the evolutionary tree represent differences between genes, and the length of the branches indicates the genetic distance between genes. This distance can be determined by the number axis on the graph, with the numbers on the axis representing amino acid substitutions.

**Figure 3:**
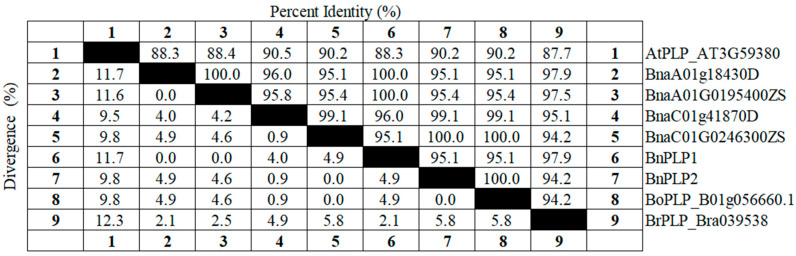
Analysis of the similarity of coding sequence between homologous genes of *PLP* in *Arabidopsis thaliana*, *Brassica rapa*, *Brassica oleracea* and three *Brassica napus* rapeseed varieties. “Percent Identity” refers to the similarity between amino acid sequences of genes. “Divergence” refers to the differences between amino acid sequences. Numbers 1 to 9, respectively, represent AtPLP_AT3G59380, BnaA01g18430D, BnaA01G0195400ZS, BnaC01g41870D, BnaC01G0246300ZS, BnPLP1, BnPLP2, BoPLP_B01g056660.1, and BrPLP_Bra039538.

By combining *AtPLP* with the homologous gene *BrPLP* (Bra039538) in *B. rapa* and the homologous gene *BoPLP* (Bo1g056660.1) in *B. oleracea*, a phylogenetic analysis of the *BnPLP1/2* genes based on amino acid sequences was conducted (Figure 2). It was found that *BnPLP1* originated from the *B. rapa* parent’s *BrPLP*, while *BnPLP2* originated from the *B. oleracea* parent’s *BoPLP* (Figure 2). The variation level (2.1%) of *BnPLP1* compared to *BrPLP* was significantly higher than the variation level (0%) of *BnPLP2* compared to *BoPLP* (Figure 3). In addition, the sequence identity of *BnPLP1* with BnaA01T0195400ZS (100%) and with BnaA01g18430D (100%) is higher than its sequence identity with BnaC01G0246300ZS (95.1%) and with BnaC01g41870D (96%) (Figure 3). On the other hand, the sequence identity of *BnPLP2* with BnaC01G0246300ZS (100%) and with BnaC01g41870D (99.1%) is higher than its sequence identity with BnaA01T0195400ZS (95.4%) and with BnaA01g18430D (95.1%) (Figure 3). This means that *BnPLP1* refers to BnaA01T0195400ZS in “ZS11” and BnaA01g18430D in “Darmor”, while *BnPLP2* refers to BnaC01G0246300ZS in “ZS11” and BnaC01g41870D in “Darmor”.

### 2.2. Gene Expression Analysis of BnPLPs

**Figure 4:**
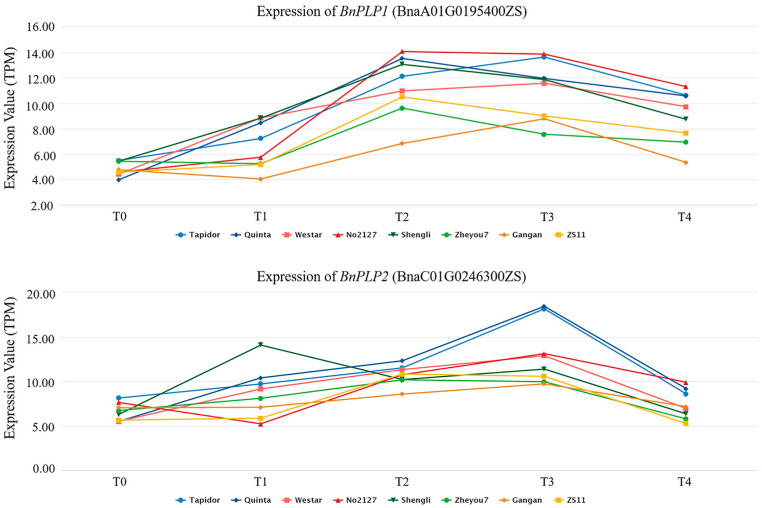
Spatial expression patterns of *BnPLP1* and *BnPLP2* genes in leaves of *Brassica napus*. T0 represents 24 days after sowing, T1 represents 54 days after sowing, T2 represents 82 days after sowing, T3 represents 115 days after sowing, and T4 represents 147 days after sowing. Eight varieties of *B. napus*, namely, “Westar”, “Quinta”, “Tapidor”, “Shengli”, “Zheyou”, “Gangan”, “ZS11”, and “No2127”, were used for this analysis.

Based on the multi-omic information resources of *B. napus* [55], the spatiotemporal expression characteristics of *BnPLP1/2* genes were obtained and visualized. From the perspective of gene expression temporal patterns, the expression levels of *BnPLP1/2* showed an increasing trend followed by a decreasing trend with the progression of growth time in eight varieties of *B. napus* leaves, namely, “Westar”, “Quinta”, “Tapidor”, “Shengli”, “Zheyou”, “Gangan”, “ZS11”, and “No2127” (Figure 4). The expression level of *BnPLP1* (BnaA01T0195400ZS) reached its maximum in the leaves at 82 days after sowing (T2), except for in the varieties “Tapidor”, “Westar”, and “Gangan”, where it was downregulated afterwards (Figure 4). On the other hand, the expression level of *BnPLP2* (BnaC01G0246300ZS) reached its maximum in the leaves at 115 days after sowing (T3), except for in the variety “Shengli”, where it was downregulated afterwards (Figure 4). Overall, the temporal expression patterns of *BnPLP1* and *BnPLP2* in different rapeseed varieties’ leaves are relatively conservative.

**Figure 5:**
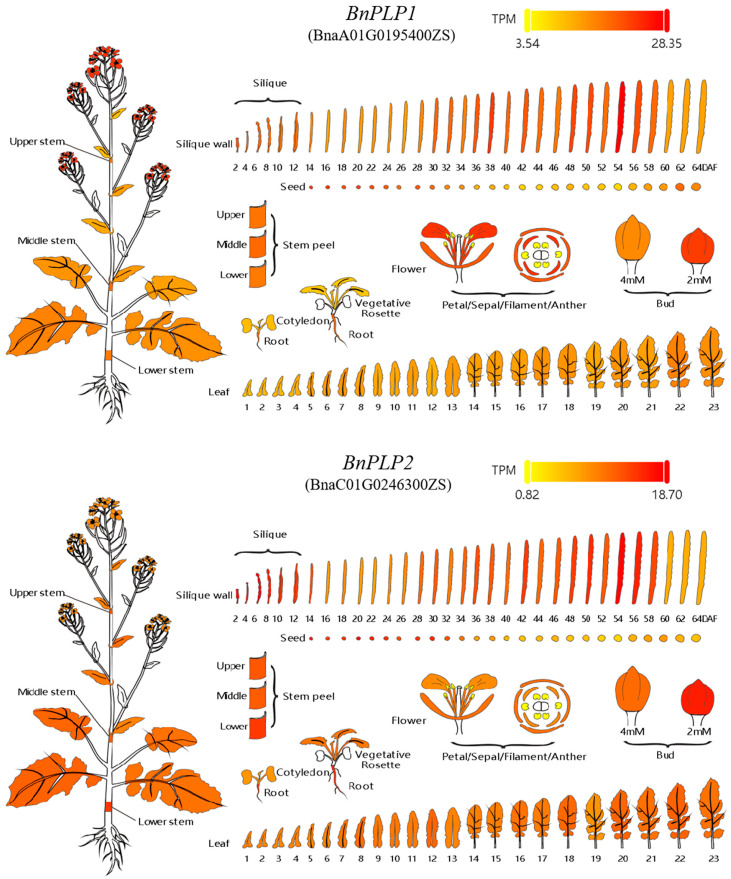
Tissue expression characteristics of *BnPLP1* and *BnPLP2* genes in *Brassica napus*. Data related to TPM, which stands for Transcripts Per Million, indicate that the higher the TPM value, the darker the color in the corresponding tissue, indicating a higher gene expression level in that tissue.

Regarding the spatial expression pattern of genes, it was shown that *BnPLP1* (BnaA01T0195400ZS) is expressed in the whole plant during the flat leaf stage of rapeseed, as well as in the leaves and roots during the seedling stage, although the expression level is relatively low (Figure 5). During the flowering period, *BnPLP1* exhibits similar expression levels in the upper, middle, and lower stem epidermis of the plants, while the expression level gradually increases from the 1st leaf (counted from the top downwards) to the 23rd leaf on the main stem. In small flower buds, the expression level of *BnPLP1* is significantly higher than in large flower buds. In open flowers, *BnPLP1* is expressed in sepals, petals, and stamens, but no gene expression of *BnPLP1* was detected in pistils. After analyzing the expression level of *BnPLP1* in pods from the second day to 64 days after flowering, it was found that the expression level of *BnPLP1* in silique pericarp was low during the early stage of silique development (within 30 days after flowering). However, the expression level significantly increased during the middle stage of silique development (from 30 to 58 days after flowering), and then decreased significantly during the late stage of silique development (from 60 to 64 days after flowering). Conversely, the expression level of *BnPLP1* in the corresponding seeds at the different stages of silique development showed an opposite trend compared to that in the silique epidermis. In addition, the tissue expression characteristics of *BnPLP2* during different developmental stages of rapeseed are similar to those of *BnPLP1*, but the expression level of *BnPLP2* in various tissues is significantly lower than that of *BnPLP1* (Figure 5).

### 2.3. Overexpression and Knockout Plants Construction of BnPLP1 Gene

In our previous study, a high expression of *BnPLP1* (BnaA01g18430D) was detected through mRNA sequencing analysis of the inflorescence (including shoot apical meristem and flower buds at stages 1 to 5) of “APL01”, while no expression of *BnPLP2* was detected [56]. Therefore, in this study, we focused solely on elucidating the functions of *BnPLP1*.

**Figure 6:**
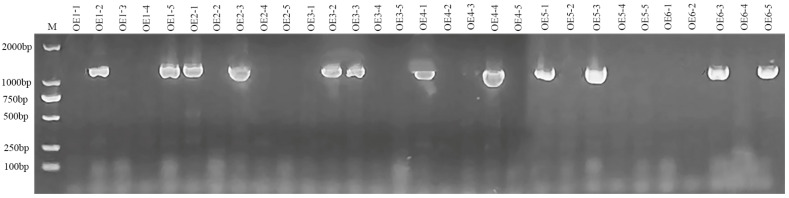
PCR identification of T2 transgenic *Arabidopsis* plants overexpressing the *BnPLP1* gene.

**Figure 7:**
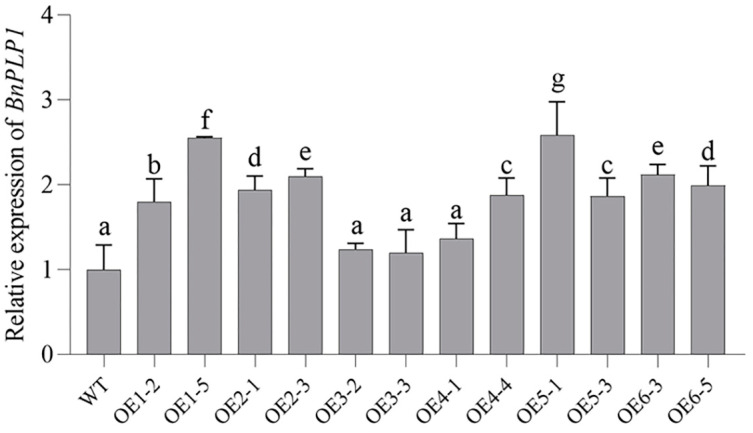
qRT-PCR identification of transgenic *Arabidopsis* T2 plants overexpressing *BnPLP1*. The lowercase letters labeled on the bar graph indicate the significance of differences in *BnPLP1* gene expression levels between genetically modified plants and wild-type.

For the creation of transgenic *Arabidopsis* plants overexpressing the *BnPLP1* gene (using OE as the plant code), PCR analysis was used to identify 12 positive second-generation transgenic (T2) plants from 30 T2 plants derived from 6 first-generation transgenic (T1) plants (selecting 5 T2 plants randomly from the T2 generation individuals of each T1 plant) (Figure 6). Two positive plants were identified among the five T2 individuals of each T1 plant. Quantitative reverse transcription PCR (qRT-PCR) analysis of the *BnPLP1* expression levels in these 12 plants revealed significant upregulation in 9 plants compared to the wild type plants (Figure 7), which were selected as positive transgenic candidate plants showing a stable overexpression of the *BnPLP1* gene. Furthermore, by using a combined approach of PCR and qRT-PCR analysis, the genotypes and expression levels of the *BnPLP1* gene in the third-generation transgenic (T3) plants of these nine positive T2 candidate plants were identified. The results indicate that the genotype (*BnPLP1*/*BnPLP1*) and overexpression of the *BnPLP1* gene in five transgenic candidate T2 *Arabidopsis* plants (i.e., OE1-5, OE2-3, OE5-1, OE5-3, and OE6-5) are stably inherited in T3 plants (Figures S2 and S3). Subsequently, the phenotypes of T3 plants derived from these five stable overexpressing *BnPLP1* T2 plants were measured.

**Figure 8:**
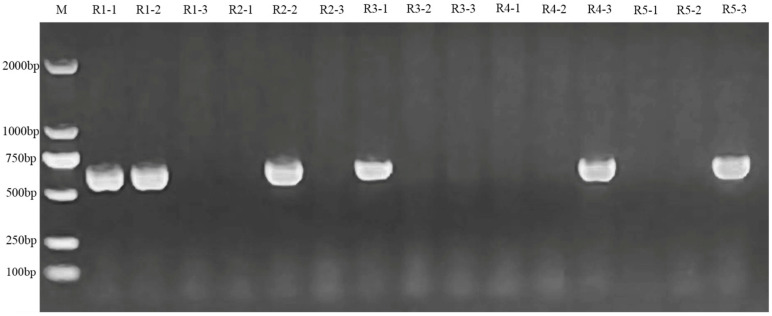
PCR identification of T2 oilseed rape plants with RNA interference targeting the *BnPLP1* gene expression.

**Figure 9:**
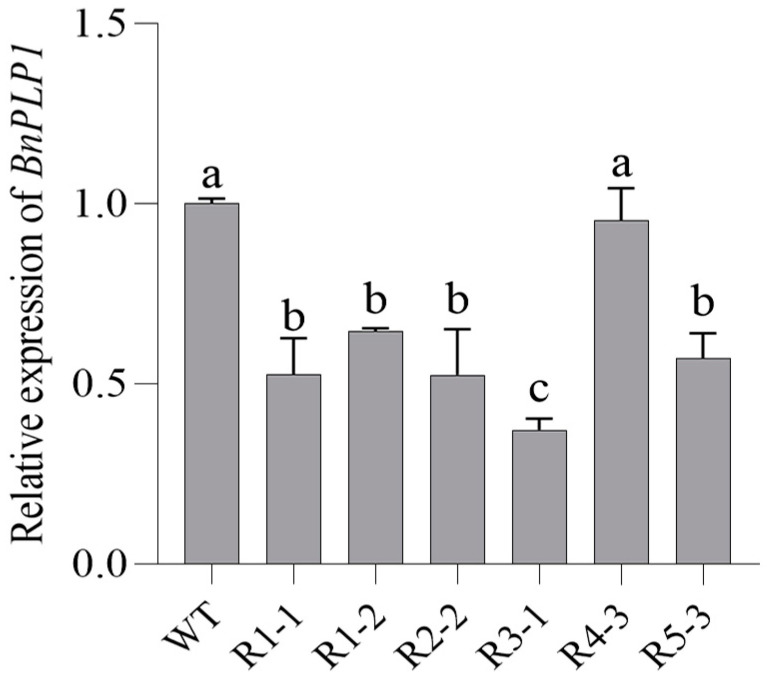
qRT-PCR identification of *BnPLP1* gene expression in RNA interference transgenic T2 plants of *Brassica napus*. The lowercase letters labeled on the bar graph indicate the significance of differences in *BnPLP1* gene expression levels between genetically modified plants and wild-type.

For the creation of RNA interference plants of *B. napus* with the downregulated expression of the *BnPLP1* gene (using R as the plant code), PCR analysis was used to screen 6 positive T2 plants from 15 T2 plants derived from 5 T1 plants (3 T2 plants were randomly selected from the T2 generation individuals of each T1 plant) (Figure 8). The qRT-PCR analysis revealed that the expression levels of *BnPLP1* in five out of the six T2 plants were significantly lower than those in the wild type (Figure 9), and these five plants were selected as stable positive transgenic candidates with *BnPLP1* silencing. Furthermore, PCR analysis and qRT-PCR analysis were performed to identify the genotypes and expression levels of the *BnPLP1* gene in T3 plants generated from these five candidate T2 plants. The results indicate that the genotype (*bnplp1*/*bnplp1*) and down-regulated expression of the *BnPLP1* gene were stably inherited in the T3 plants of three rapeseed positive T2 candidates (namely, R1-1, R2-2, and R3-1) (Figures S4 and S5). Subsequently, the phenotype of the T3 plants derived from these three T2 plants expressing downregulated *BnPLP1* was determined.

### 2.4. BnPLP1 Positively Regulates Early Flowering in Arabidopsis and Rapeseed, but Prolongs the Flowering Period

In five transgenic *Arabidopsis* plants (OE1-5, OE2-3, OE5-1, OE5-3, and OE6-5) with the stable overexpression of *BnPLP1*, the flowering initiation dates of all plants were significantly earlier than those of wild type plants (Table 1). Regarding the flowering termination dates, two plants (OE5-1 and OE6-5) showed a delay compared to the wild type, one plant (OE2-3) displayed an earlier termination, and the remaining two plants (OE1-5 and OE5-3) showed no significant differences compared to the wild type (Table 1). Furthermore, the flowering period of all five transgenic plants was longer than that of the wild type (Table 1).

In oilseed rape, the flowering initiation of three *BnPLP1* downregulated plants (R1-1, R2-2 and R3-1) was significantly delayed compared to the wild type plants (Table 2). Except for R1-1, which showed delayed flowering termination, there were no significant differences in the flowering termination between the other two plants, R2-2 and R3-1, and the wild type (Table 2). From the perspective of flowering period, the three *BnPLP1* downregulated genotypes exhibited a significantly short flowering period compared to the wild type (Table 2).

Observations on the morphology of floral organs reveal that *B. napus* plants with the downregulated expression of *BnPLP1* did not exhibit significant changes in the numbers and sizes of floral organs compared to the wild type plants (Figure S6). This is completely different from the floral organ phenotype of the *Arabidopsis plp* mutant. It cannot be ruled out that *BnPLP1* has lost its regulatory role in controlling the morphogenesis of floral organs during evolution.

### 2.5. BnPLP1 Positively Regulates Plant Height and Main Inflorescence Length of Rapeseed

**Figure 10:**
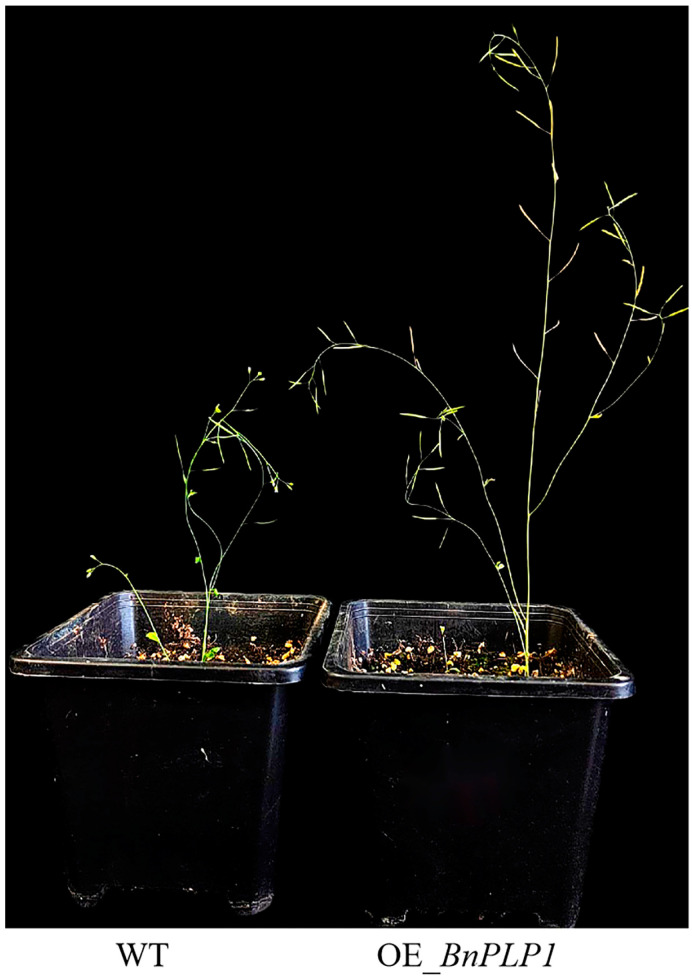
The effect of the stable overexpression of the *BnPLP1* gene on plant height and main inflorescence length in Colombian wild type *Arabidopsis thaliana*. WT refers to wild type *Arabidopsis thaliana* plants. OE_*BnPLP1* represents *Arabidopsis thaliana* plants overexpressing the *BnPLP1* gene.

Comparing the plant height, main inflorescence length, and primary branching number between genetically modified *Arabidopsis thaliana* (GM) and wild type plants, it was found that the plant height and length of the main inflorescence were significantly higher in five GM plants overexpressing the *BnPLP1* gene (OE1-5, OE2-3, OE5-1, OE5-3, and OE6-5) compared to the wild type (Figure 10, Table 3). However, there was no significant difference in the number of primary branches between the GM and wild type plants (Table 3).

**Figure 11:**
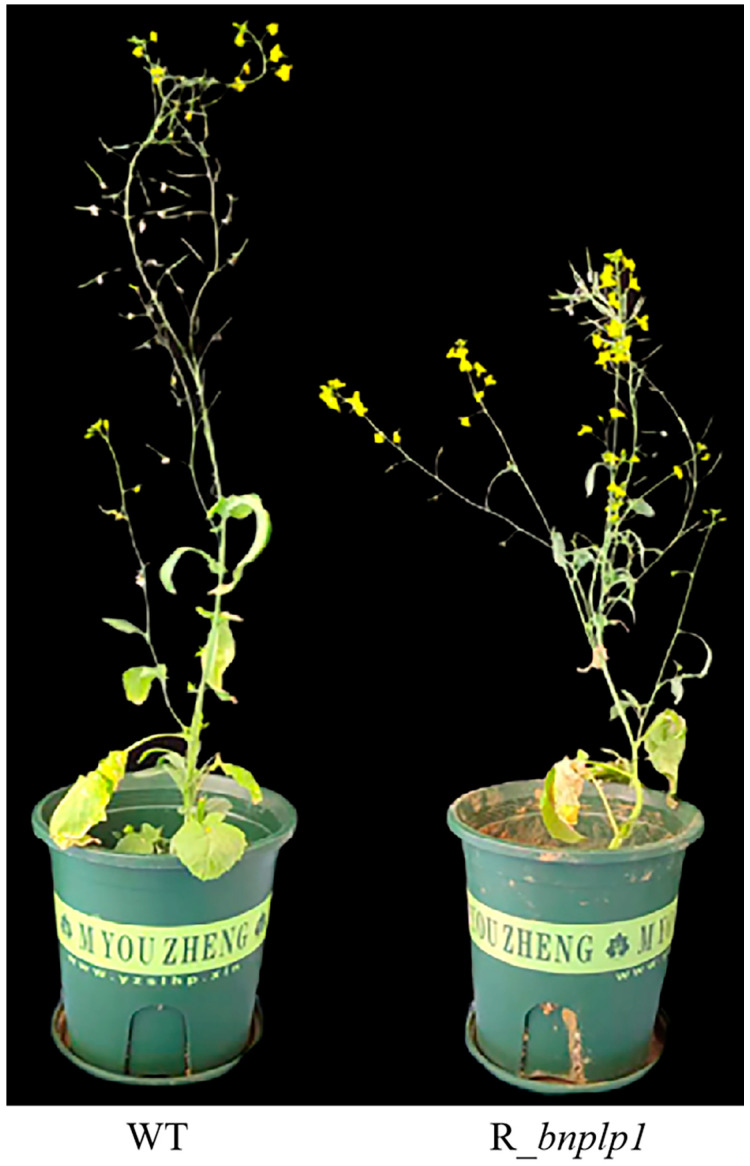
Effects of the stable downregulation of *BnPLP1* gene expression on plant height and main inflorescence length in *Brassica napus*. WT refers to wild type *Brassica napus* plants. R_*bnplp1* represents *Brassica napus* plants with the expression of the *BnPLP1* gene downregulated by RNA interference.

**Figure 12:**
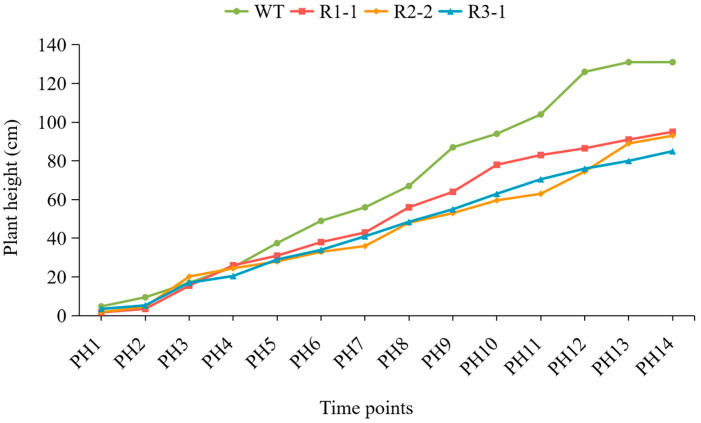
Growth dynamics of the plant height in stable downregulated *BnPLP1*-expressing *Brassica napus* plants and wild type plants. WT refers to wild type *Brassica napus* plants. R represents *Brassica napus* plants with expression of the *BnPLP1* gene downregulated by RNA interference. PH1: 12 days after sowing; PH2: 19 days after sowing; PH3: 26 days after sowing; PH4: 33 days after sowing; PH5: 40 days after sowing; PH6: 47 days after sowing; PH7: 54 days after sowing; PH8: 61 days after sowing; PH9: 68 days after sowing; PH10: 75 days after sowing; PH11: 82 days after sowing; PH12: 89 days after sowing; PH13: 96 days after sowing; PH14: 103 days after sowing.

**Figure 13:**
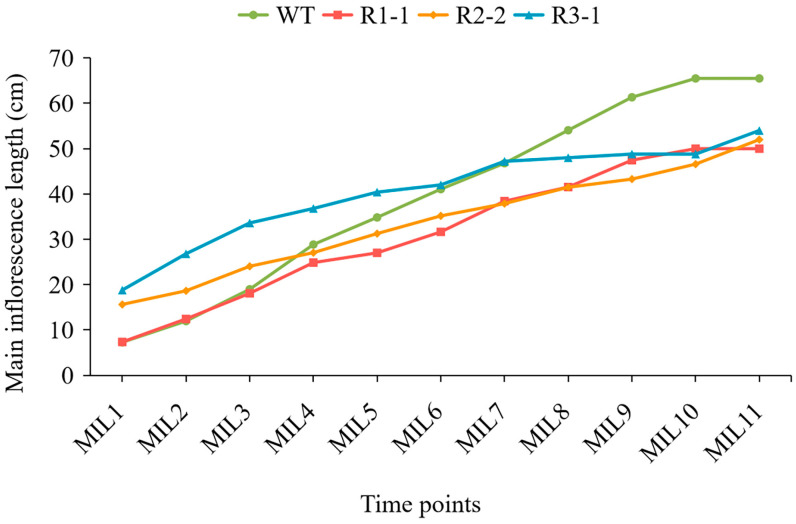
Growth dynamics of the main inflorescence length in stable downregulated *BnPLP1*-expressing *Brassica napus* plants and wild type plants. The definitions of WT and R are consistent with those in Figure 12. MIL1: 33 days after sowing; MIL2: 40 days after sowing; MIL3: 47 days after sowing; MIL4: 54 days after sowing; MIL5: 61 days after sowing; MIL6: 68 days after sowing; MIL7: 75 days after sowing; MIL8: 82 days after sowing; MIL9: 89 days after sowing; MIL10: 96 days after sowing; MIL11: 103 days after sowing.

In rapeseed, the plant heights and main inflorescence lengths of three *BnPLP1* RNAi plants (R1-1, R2-2 and R3-1) were significantly reduced compared to the wild type (Figure 11, Table 4). However, there was no significant difference in the number of primary branches and stem diameter between the RNAi and wild type plants (Table 4). Further analysis of the growth dynamics revealed that the height of wild type plants rapidly increased starting from the fifth measurement (referred to as PH5), while the height of the three *BnPLP1* silenced plants grew slowly (Figure 12). Consequently, the difference in plant height between RNAi and wild type plants gradually increased (Figure 12). This indicates that although *BnPLP1* is overexpressed throughout the entire growth and development period of rapeseed, its regulatory role in controlling the plant height of rapeseed can only be exerted at specific time points. From the growth dynamics of the main inflorescence length, two RNAi plants (R1-1 and R2-2) have consistently shown significantly shorter main inflorescence lengths compared to the wild type, starting from the fifth measurement for main inflorescence length (referred to as MIL5) and reaching until the main inflorescence stops elongating (referred to as MIL11) (Figure 13). The main inflorescence length of another RNAi plant, R3-1, was significantly lower than that of the wild type starting from the eighth measurement (referred to as MIL8) (Figure 13). This indicates that the time point at which *BnPLP1* regulates the elongation of the main inflorescence is significantly later than the time point at which it regulates plant height.

## 3. Discussion

The flowering time, plant height, and length of the main inflorescence in rapeseed are quantitative traits controlled by multiple genes [8,31,32,33,34,35,36,37,38,39,40,41,42,43,44,57,58]. Protein prenylation is involved in plant growth, development, and environmental responses [51,52]. Previous studies have shown that protein prenylation mediated by PFT and PGGT-I is involved in the regulation of multiple traits such as plant growth, meristem size, flowering time, plant height, stem morphology, and number of floral organs in *Arabidopsis* [48]. *PLP*, as the encoding gene of the common α-subunit in PFT and PGGT-I, has only one copy in *Arabidopsis* [48]. Based on the evolutionary relationship between rapeseed and *Arabidopsis* [2], it can be inferred that there are at least two homologous genes of *PLP* in rapeseed. In this study, through CDS alignment in the genomic databases of “Darmor” and “ZS11”, and using “APL01” as a template for gene cloning, it was confirmed that two homologous genes of *PLP*, *BnPLP1* and *BnPLP2*, do exist in *B. napus*. Evolutionary analysis revealed that *BnPLP1* originated from *B. rapa* and exhibited larger sequence variations, while *BnPLP2* originated from *B. oleracea* with smaller sequence variations. This once again indicates that during the evolution process of *B. napus*, the A genome underwent larger sequence variations compared to the C genome [2].

Gene expression analysis indicates that the spatial and temporal expression patterns of *BnPLP1* and *BnPLP2* are similar, but the expression level of *BnPLP2* is consistently lower than that of *BnPLP1*. This suggests that *BnPLP1* plays a major role in numerous growth- and development-related traits in rapeseed. In fact, the previous mRNA sequencing of rapeseed inflorescences only detected the expression of *BnPLP1* [56]. In another study, Wang et al. cloned only one *BnPLP* gene (also known as *BnFTA*) from *B. napus* and assumed that there was only one active form of *BnPLP* in rapeseed [54]. When combining the results of the tissue-specific expression analysis of *BnPLP1* and *BnPLP2* in this study, it is speculated that this is likely due to the relatively lower expression level of *BnPLP2* compared to *BnPLP1*. These studies also indirectly suggest that *BnPLP1* may play a more critical role than *BnPLP2* in the growth and development of rapeseed.

The results of this study show that the overexpression of *BnPLP1* promotes early flowering in *Arabidopsis*, while the downregulation of its expression in rapeseed delays flowering. Thus, it can be concluded that *BnPLP1* plays a role in promoting early flowering in plants. This is consistent with the function of *PLP* in *A. thaliana* [48], indicating that the regulatory function of the *PLP* gene in promoting early flowering in plants is conserved between these two species. From the perspective of the flowering period, the upregulation of *BnPLP1* expression also has the function of prolonging the flowering period of *B. napus*. This may be due to the advancement of initial flowering, as changes in *BnPLP1* expression do not significantly alter the final flowering time of plants. The mechanism of *AtPLP* regulation on flowering time in *A. thaliana*, as suggested by Running et al. [48], is highly likely to be through the regulation of plant growth rate. In this study, based on the comparison of the flowering times of transgenic plants and wild types, both the upregulation and the downregulation of *BnPLP1* expression did not significantly affect plant growth rate, indicating that *BnPLP1* may be involved in the regulation of flowering time through a different pathway than *AtPLP*.

In addition to flowering time, the plant height and main inflorescence length are the traits most significantly influenced by *BnPLP1* gene expression in *A. thaliana* and *B. napus*. The results of this study demonstrate that the upregulation of *BnPLP1* expression significantly increases plant height and main inflorescence length in *Arabidopsis*, whereas the downregulation of expression reduces plant height and main inflorescence length in rapeseed. This indicates that *BnPLP1* plays a positive regulatory role in these two traits. The positive regulatory effect of *BnPLP1* on the plant height of rapeseed is consistent with the function of *AtPLP* in *Arabidopsis* [48]. From the dynamics of plant height and main inflorescence length, it can be observed that *BnPLP1* exhibits a noticeable time lag between regulating the plant height and main inflorescence length of rapeseed. This may be attributed to the time-specific expressions of genes downstream of *BnPLP1*, which play a regulatory role in plant height and main inflorescence length.

It is worth mentioning that the phenotypes of the mutated oilseed plants under *BnPLP1* expression reduction were not as numerous as those in *plp Arabidopsis* mutants [48]. Furthermore, even the altered flowering time and plant height mutations in oilseed did not exhibit the same severity as in *Arabidopsis* [48]. This may be due to a dosage effect of gene expression on phenotype regulation, since the expression of the *BnPLP1* gene in the three RNAi oilseed plants in this study, although significantly reduced compared to the wild type, did not show as much reduction as *PLP* in *plp Arabidopsis* mutants. Coincidentally, a study on wheat starch synthesis showed that the gene dosage effect significantly influences plant phenotypes [59]. Another explanation for the phenotypic differences between *bnplp1* rapeseed plants and *plp Arabidopsis* plants is the evolutionary changes in the functionality of the *BnPLP1* gene compared to *AtPLP*. In fact, based on the gene expression characteristics of *BnPLP1*, it can be inferred that its function has changed compared to *AtPLP*. For example, this study found that *BnPLP1* was not expressed in the pistils of rapeseed, implying that *BnPLP1* is not involved in pistil development. Subsequent findings from this study confirmed this inference. However, in *Arabidopsis*, mutations in *AtPLP* led to an increase in pistil number [48].

Because the downregulation of *BnPLP1* expression in *B. napus* only resulted in a shorter flowering period and an appropriate reduction in plant height (approximately 31% lower than the wild type), without any other obvious detrimental effects on the growth and development of *B. napus*, it is feasible to consider using genetic engineering technology to create short-flowering and semi-dwarf *B. napus* germplasms for oilseed crop breeding. In addition, in future research, protein isoprenylation modification omics studies can be considered to explore new genes that regulate flowering time and plant architecture downstream of *BnPLP1*, in order to provide more novel genes for rapeseed breeding.

## 4. Materials and Methods

### 4.1. Plant Materials

A semi-winter type *B. napus* variety, “APL01” [60], was used for *BnPLPs* gene cloning because previous studies have shown that “APL01” exhibits early flowering and high expression of the *BnPLP* gene in this variety [56]. The GenBank accession number of *AtPLP* in NCBI is NC_003074.8, and its accession number in the *Arabidopsis* Information Resource is AT3G59380. The complete genomic DNA sequence, CDS, and amino acid sequence of *AtPLP* were obtained based on these accession numbers. Genetic transformation was carried out using Columbia ecotype (Col-0) *Arabidopsis* and a spring ecotype *B. napus* variety “862” (bred by the Oil Crops Research Institute of the Chinese Academy of Agricultural Sciences) as the receptor plants. All plant materials are cultivated in a controlled environment chamber (with 16 h of light and 8 h of darkness at a constant temperature of 25 °C, with a relative humidity of 80%).

### 4.2. DNA, Total RNA Extraction, and cDNA Synthesis

The inflorescences containing young buds at different developmental stages were taken from “APL01” for DNA and total RNA extraction. DNA was extracted using the EasyPure^®^ Plant Genomic DNA Kit (TransGen Biotech, Beijing, China), and DNA contamination was evaluated using a 1% agarose gel electrophoresis at low concentrations. Total RNA was isolated using an E.Z.N.A.^®^ Plant RNA Kit (Omega Bio-Tek, Connecticut, CT, USA), whereas RNA degradation and contamination were monitored on 1% agarose gels. The purity and concentration of the RNA were determined using a Biodrop μLite+ micro-volume spectrophotometer (Biodrop, New York, USA). Using the Biodrop μLite+ micro-volume spectrophotometer equipped with DNA/RNA standard samples, we calibrated the instrument. Then, we took 2 μL of total RNA solution and placed it in the sample slot. Finally, we read the RNA concentration, OD260/280 ratio, and 260/230 ratio. A ratio within the range of 1.8 to 2.0 indicates high RNA purity, meeting the requirements for subsequent experiments. cDNA synthesis was performed using the M-MLV first strand cDNA Synthesis Kit TQ2501 (Omega Bio-Tek).

### 4.3. Sequence Analysis

The CDS values of the *PLP* gene in *B. napus*, *B. rapa*, and *B. oleracea* were retrieved by conducting a blast search against the reference genome databases of *B. napus* [2,61], *B. rapa* [62], and *B. oleracea* [63] using the CDS of *AtPLP* [48] as a reference. At the same time, the amino acid sequences of the proteins encoded by these *PLP* homologous genes were also obtained from these reference genome databases. These sequences were aligned by Clustal-W (University College Dublin, Dublin, Ireland), and a neighbor-joining tree was built using MEGA version 4.0 [64]. BnPLP1 and BnPLP2 protein domain analysis was performed using the Pfam domains database (https://www.ebi.ac.uk/interpro/) (accessed on 1 September 2023).

### 4.4. Gene Cloning and Plasmid Construction

*BnPLP1* and *BnPLP2* were amplified from the DNA of “APL01” using two pairs of primers (PR1 and PR2) and 2×TransTaq^®^ High-Fidelity (HiFi) PCR SuperMix I (-dye) (TransGen Biotech). The thermocycler program comprised a 5-min incubation at 94 °C, followed by 35 cycles consisting of a 30-s denaturation at 94 °C, a 30-s annealing at 58 °C, and a 2-min extension at 72 °C. Subsequently, the program concluded with a 10-min final extension step at 72 °C. The CDS values of *BnPLP1* and *BnPLP2* were amplified from the cDNA of “APL01” using two pairs of primers (PR3 and PR4), and the amplification was carried out with 2×TransTaq^®^ High-Fidelity (HiFi) PCR SuperMix I (-dye) (TransGen Biotech). The thermocycler program consisted of 5 min at 94 °C, 35 cycles of 30 s at 94 °C, 30 s at 60 °C, and 1 min at 72 °C followed by 10 min at 72 °C. All primers used in this study were designed using the Primer 5 software and synthesized by Tsingke Biotechnology Co. (Beijing, China). The sequences of these primers are listed in Table S1. After that, the PCR product was cloned into the pEASY^®^-T1 cloning vector (TransGen Biotech), followed by transformation into *Escherichia coli* competent cells (Trans1-T1 Phage Resistant Chemically Competent Cell, TransGen Biotech), and finally subjected to sequencing using primers M13F 5′-GTAAAACGACGGCCAGT-3′ and M13R 5′-CAGGAAACAGCTATGAC-3′. The alignment of DNA and CDS sequences between these two genes was performed using Clustal-W.

The correct cloning of CDS sequence was recombined (PR5) into the modified pCAMBIA1303 vector [65] to create a constitutive overexpression (hereafter called OE) vector of the *BnPLP1* gene driven by the constitutive Caulifower mosaic virus (CaMV) 35S promoter using the pEASY^®^-Basic Seamless Cloning and Assembly Kit (TransGen Biotech). The restriction endonuclease site *EcoR*I was chosen to linearize the pCAMBIA1303 vector. The glycine promoter on the expression vector pCAMBIA1303 was replaced by the CaMV 35S promoter. According to the product manual of the pEASY^®^-Basic Seamless Cloning and Assembly Kit, the homologous recombination of *BnPLP1* CDS with linearized vector pCAMBIA1303 was performed. A pair of primers (PR6) were used to verify the successful recombination of the CDS of *BnPLP1* with linearized pCAMBIA1303.

A fragment of 435 bp from the CDS of the *BnPLP1* gene was cloned from the cDNA of “APL01” using the primer PR7. This fragment was then ligated (PR7) into the pEASY^®^-T1 cloning vector (TransGen Biotech) and transformed into *E. coli* competent cells (TransGen Biotech) for sequencing (M13F 5′-GTAAAACGACGGCCAGT-3′ and M13R 5′-CAGGAAACAGCTATGAC-3′). The correctly sequenced RNAi fragments in sense and antisense strands were sequentially recombined (PR8 and PR9) into the modified pCAMBIA1390 vector [65] using the pEASY^®^-Basic Seamless Cloning and Assembly Kit (TransGen Biotech) to construct an RNAi vector for silencing the expression of the *BnPLP1* gene. The restriction endonuclease sites *Sac*I and *SnaB*I were selected sequentially for linearizing the pCAMBIA1390 vector, and then used consecutively for recombination with the sense and antisense strands of the RNAi fragment. Primers PR10 and PR11 were used to confirm the successful recombination of the sense and antisense strands of the RNAi fragment with linearized pCAMBIA1390.

### 4.5. Plant Transformation and Positive Plant Identification

The OE vector and RNAi vector of the *BnPLP1* gene were transformed into the *Agrobacterium tumefaciens* strain GV3101. *A. tumefaciens* carrying the OE vector was used to infect Columbia ecotype *A. thaliana* to generate a plant overexpressing the *BnPLP1* gene, while *A. tumefaciens* carrying the RNAi vector was used to infect the “862” variety of *B. napus* to generate a rapeseed plant with silenced *BnPLP1* gene expression. The floral dipping method [66] was employed to transform *Arabidopsis* plants. Seeds were harvested and screened on 0.8% agar plates containing ½ MS and 25 mg L^−1^ of hygromycin. The total DNA was extracted from the young leaves of T1 plants using the MagicPure^®^ Plant Genomic DNA Kit (TransGen Biotech). PCR was used to determine the presence of the *BnPLP1* gene using a pair of validation primers (PR6). The hypocotyl of *B. napus* variety “862” was utilized for *Agrobacterium*-mediated transformation, following the protocol described by Zhang et al. [65]. Primer PR10 was used to identify positive transgenic *B. napus* plants transformed with RNAi fragments. Positive transgenic plants were bagged for self-pollination, and the mature seeds were harvested for subsequent experiments.

### 4.6. Gene Expression Analysis

Regarding the spatiotemporal expression characteristics of the *BnPLP1/2* genes, the IDs (BnaA01T0195400ZS and BnaC01G0246300ZS) of *BnPLP1* and *BnPLP2* genes in “ZS11” were separately inputted into the “Transcriptomics” menu of the multi-omics information database for *B. napus* (https://yanglab.hzau.edu.cn/) (accessed on 9 September 2023). Upon submission, the spatiotemporal expression pattern information of these two genes was obtained online, and visual images were generated.

PCR-identified positive *Arabidopsis* and *B. napus* T2 and T3 plants’ young leaves were collected for total RNA extraction using the E.Z.N.A.^®^ Plant RNA Kit (Omega Bio-Tek). RNA degradation and contamination were monitored on 1% agarose gels. The purity and concentration of the RNA were determined using a Biodrop μLite+ micro-volume spectrophotometer (Biodrop). Subsequently, cDNA synthesis was performed using HiScript II Q Select RT SuperMix for qPCR(+gDNA wiper) (Vazyme, Nanjing, China). The gene-specific primer PR12 and Taq Pro Universal SYBR qPCR Master Mix (Vazyme) were used for quantitative real-time PCR (qPCR) assays of the *BnPLP1* gene using the BIO-RAD CFX96 Real-Time PCR System (BIO-RAD, California, USA). The *B. napus* actin gene (*BnACT2*) [67] (PR13) and the *Arabidopsis* actin gene (*AtACT7*) [68] (PR14) were used as internal reference genes, and triplicate quantitative assays were performed on each cDNA dilution. The synthetic cDNA of *Arabidopsis* and *B. napus* T2 and T3 plants mentioned above were used as templates for qPCR experiments. The relative expression level of each gene was estimated using the 2^−∆∆Ct^ method [69].

### 4.7. Phenotypic Determination

Wild type and all transgenic plants were grown in a light incubator with 16 h of light and 8 h of darkness at a constant temperature of 25 °C. For *Arabidopsis*, the onset of flowering, end of flowering, flowering period, plant height, main inflorescence length, primary branching, and number and size of floral organs were measured in 5 wild type plants and 25 T3 plants derived from 5 stable *BnPLP1*-overexpressing T2 plants (5 plants randomly measured from the T3 generation individuals of each T2 plant). In the rapeseed plants, onset of flowering, the end of flowering, flowering period, plant height, main inflorescence length, number of primary branches, stem diameter, and number and size of floral organs were measured in 5 wild type plants and 15 T3 plants generated from 3 T2 plants expressing downregulated *BnPLP1* (we measured 5 plants randomly selected from the T3 generation individuals of each T2 plant). The method proposed by Yu et al. [57] was used to measure the characteristics related to flowering time. The number of days from sowing seeds to the initiation of flowering in plants was referred to as the onset of flowering. The number of days from sowing seeds to the termination of flowering in plants was referred to as the end of flowering. The number of days from the initiation of flowering to the termination of flowering in plants was referred to as the flowering period. From the 12th day after sowing, transgenic plants showed significant differences in plant height compared to wild type plants. We measured every seven days until there was no significant increase, in order to determine the dynamic characteristics of plant height changes. The specific method used was derived from Wang et al. [70] with slight modifications. According to the method of Zheng et al. [40], the number of primary branches was counted. The stem diameter was measured following the method of Yu et al. [71]. The length of the main inflorescence was measured according to the method of Chen et al. [42]. We measured the length of the main inflorescence every seven days from 33 days after sowing until the length of the main inflorescence was no longer visibly increasing. Twenty fully open flowers on each plant were randomly selected to measure the number and size of floral organs. The calculation of the mean and standard deviation, as well as the comparison between means, was performed using SAS v9.4 software (SAS Institute Inc., North Carolina, USA). The unpaired *t*-test method was employed to analyze the significance of differences between pairwise data.

## 5. Conclusions

In this study, bioinformatics analysis and gene cloning demonstrated the existence of two homologous genes of *AtPLP* in the *B. napus* genome, namely, *BnPLP1* and *BnPLP2*. Evolutionary analysis revealed that *BnPLP1* originated from the *B. rapa* genome, exhibiting significant sequence variation compared to *BrPLP*, while *BnPLP2* originated from the *B. oleracea* genome, showing relatively minor sequence variation compared to *BoPLP*. By constructing *BnPLP1*-overexpressing *A. thaliana* plants and downregulating *BnPLP1* expression in *B. napus* plants, the role of *BnPLP1* in promoting early flowering, prolonging the flowering period, as well as positively regulating plant height and main inflorescence length has been elucidated. These results help to reveal the biological role of protein prenylation in *B. napus*. Taking into account the benefits brought by the downregulation of the *BnPLP1* gene in rapeseed, it can potentially be used in rapeseed breeding to create short-flowering period, semi-dwarf rapeseed germplasms through genetic engineering methods.

## Figures and Tables

**Table 1:** Comparison of flowering time-related traits between transgenic *Arabidopsis* plants overexpressing the *BnPLP1* gene and wild type plants.

Genotype	Onset of Flowering	End of Flowering	Flowering Period
WT	26.2 ± 0.4 a	50.4 ± 0.5 a	24.2 ± 0.4 a
OE1-5	21.6 ± 0.5 b	50.8 ± 0.5 a	29.2 ± 0.4 b
OE2-3	21.4 ± 0.5 b	48.0 ± 0.7 b	26.6 ± 0.5 b
OE5-1	24.4 ± 0.5 b	52.6 ± 0.5 b	28.2 ± 0.4 b
OE5-3	21.2 ± 0.5 b	50.4 ± 0.5 a	29.2 ± 0.4 b
OE6-5	22.8 ± 0.8 b	52.0 ± 0.7 b	29.2 ± 0.4 b
OE_mean	22.3 ± 1.3 b	50.8 ± 1.8 a	28.5 ± 1.1 b

WT refers to wild type *Arabidopsis thaliana* plants. OE represents *Arabidopsis thaliana* plants overexpressing the *BnPLP1* gene. The unpaired *t*-test method was employed to analyze the significance of differences between pairwise data. Letters in the table represent the significance of differences between each pair of data.

**Table 2:** Comparison of flowering time-related traits between RNA-interfered *Brassica napus* plants with downregulated *BnPLP1* gene expression and wild type plants.

Genotype	Onset of Flowering	End of Flowering	Flowering Period
WT	23.8 ± 0.8 a	53.2 ± 1.3 a	29.4 ± 1.1 a
R1-1	27.8 ± 0.8 b	55.2 ± 0.8 b	27.4 ± 0.8 b
R2-2	27.6 ± 1.1 b	54.8 ± 0.8 a	27.2 ± 0.9 b
R3-1	28.0 ± 0.7 b	54.0 ± 0.7 a	26.0 ± 0.7 b
R_mean	27.8 ± 0.2 b	54.7 ± 0.6 a	26.9 ± 0.8 b

WT refers to wild type *Brassica napus* plants. R represents *Brassica napus* plants with downregulated expression of *BnPLP1* gene by RNA interference. The unpaired *t*-test method was employed to analyze the significance of differences between pairwise data. Letters in the table represent the significance of differences between each pair of data.

**Table 3:** Comparison of plant height, main inflorescence length, and number of primary branches between transgenic *Arabidopsis* plants overexpressing the *BnPLP1* gene and wild type plants.

Genotype	Plant Height	Main Inflorescence Length	Number of Primary Branches
WT	27.9 ± 1.4 a	16.4 ± 1.1 a	3.2 ± 0.4 a
OE1-5	36.0 ± 1.6 b	24.0 ± 1.6 b	3.8 ± 0.4 a
OE2-3	34.8 ± 1.3 b	23.8 ± 1.3 b	2.8 ± 0.4 a
OE5-1	35.4 ± 1.7 b	23.4 ± 1.5 b	3.0 ± 0.4 a
OE5-3	35.0 ± 1.6 b	23.8 ± 1.3 b	3.2 ± 0.4 a
OE6-5	37.6 ± 1.1 b	25.6 ± 2.1 b	3.2 ± 0.4 a
OE_mean	35.8 ± 1.1 b	24.1 ± 0.9 b	3.2 ± 0.4 a

The definitions of WT and OE are consistent with those in Table 1. The unpaired *t*-test method was employed to analyze the significance of differences between pairwise data. Letters in the table represent the significance of differences between each pair of data.

**Table 4:** Comparison of plant height, main inflorescence length, primary branching number, and stem thickness between RNA interference knockdown *BnPLP1* gene expression *Brassica napus* plants and wild type plants.

Genotype	Plant Height	Main Inflorescence Length	Number of Primary Branches	Stem Diameter
WT	131.6 ± 2.7 a	65.4 ± 1.1 a	6.2 ± 0.4 a	1.3 ± 0.1 a
R1-1	91.4 ± 2.1 b	50.0 ± 1.5 b	6.8 ± 0.4 a	1.2 ± 0.1 a
R2-2	92.8 ± 1.3 b	52.0 ± 0.7 b	7.0 ± 0.7 a	1.3 ± 0.1 a
R3-1	86.6 ± 2.1 b	54.0 ± 1.6 b	7.2 ± 0.8 a	1.2 ± 0.1 a
R_mean	90.3 ± 3.3 b	52.0 ± 2.0 b	7.0 ± 0.2 a	1.2 ± 0.1 a

The definitions of WT and R are consistent with those in Table 2. The unpaired *t*-test method was employed to analyze the significance of differences between pairwise data. Letters in the table represent the significance of differences between each pair of data.

## Data Availability

The data presented in this study are available in article or Supplementary Material.

## Supplementary Materials

The following supporting information can be downloaded at: https://www.mdpi.com/article/10.3390/genes14122206/s1, Figure S1: Structural domain analysis of (A) BnPLP1 and (B) BnPLP2 proteins; Figure S2: PCR identification of T3 generation plants derived from five stable T2 plants overexpressing the *BnPLP1* gene; Figure S3: qRT-PCR identification of T3 generation plants derived from five stable T2 plants overexpressing the *BnPLP1* gene. The x-axis represents T3 individuals of five stable transgenic plants expressing the *BnPLP1* gene, and the y-axis represents the relative gene expression level; Figure S4: PCR identification of T3 generation plants derived from stable T2 plants showing downregulated expression of the *BnPLP1* gene; Figure S5: qRT-PCR identification of T3 generation plants derived from stable T2 plants showing downregulated expression of the *BnPLP1* gene. The x-axis represents the T3 generation individuals of T2 plants with stable downregulation of the *BnPLP1* gene expression, while the y-axis represents the relative gene expression level; Figure S6: The effect of stable down-regulation of *BnPLP1* gene expression on the number and size of flower organs in *Brassica napus*. Bar, 1 cm; Table S1: The complete primer list used in this study; Supplementary File S1: DNA sequence alignment analysis between *BnPLP1* and *BnPLP2* genes; Supplementary File S2: CDS alignment analysis between *BnPLP1* and *BnPLP2* genes.

## References

[1] Liu M., Chang W., Yu M., Fan Y., Shang G., Xu Y., Niu Y., Liu X., Zhu H., Dai L. (2021). Overexpression of *DEFECTIVE IN ANTHER DEHISCENCE 1* increases rapeseed silique length through crosstalk between JA and auxin signaling. Ind. Crops Prod..

[2] Chalhoub B., Denoeud F., Liu S., Parkin I.A.P., Tang H., Wang X., Chiquet J., Belcram H., Tong C., Samans B. (2014). Early allopolyploid evolution in the post-Neolithic *Brassica napus* oilseed genome. Science.

[3] Michaels S.D. (2009). Flowering time regulation produces much fruit. Curr. Opin. Plant Biol..

[4] Fu T. (2008). On research and application of heterosis in rapeseed. Chin. J. Oil Crop Sci..

[5] Fu T., Zhou Y. (2013). Progress and future development of hybrid rapeseed in China. Eng. Sci..

[6] Chen J., Cai L., Yu K. (2022). Variation and correlation analysis of six agronomic traits in *Brassica napus* germplasm population. J. Mt. Agric. Biol..

[7] Khan S., Anwar S., Kuai J., Noman A., Shahid M., Din M., Ali A., Zhou G. (2018). Alteration in yield and oil quality traits of winter rapeseed by lodging at different planting density and nitrogen rates. Sci. Rep..

[8] Wellmer F., Riechmann J.L. (2010). Gene networks controlling the initiation of flower development. Trends Genet..

[9] Amasino R. (2010). Seasonal and developmental timing of flowering. Plant J..

[10] Geraldo N., Baurle I., Kidou S., Hu X., Dean C. (2009). *FRIGIDA* delays flowering in *Arabidopsis* via a cotranscriptional mechanism involving direct interaction with the nuclear cap-binding complex. Plant Physiol..

[11] Kim D.H., Doyle M.R., Sung S., Amasino R.M. (2009). Vernalization: Winter and the timing of flowering in plants. Annu. Rev. Cell Dev. Biol..

[12] Wang J., Long Y., Wu B., Liu J., Jiang C., Shi L., Zhao J., King G.J., Meng J. (2009). The evolution of *Brassica napus FLOWERING LOCUS T* paralogues in the context of inverted chromosomal duplication blocks. BMC Evol. Biol..

[13] Hou J., Long Y., Raman H., Zou X., Wang J., Dai S., Xiao Q., Li C., Fan L., Liu B. (2012). A Tourist-like MITE insertion in the upstream region of the *BnFLC.A10* gene is associated with vernalization requirement in rapeseed (*Brassica napus* L.). BMC Plant Biol..

[14] Zou X., Suppanz I., Raman H., Hou J., Wang J., Long Y., Jung C., Meng J. (2012). Comparative analysis of *FLC* homologues in *Brassicaceae* provides insight into their role in the evolution of oilseed rape. PLoS ONE.

[15] Tadege M., Sheldon C.C., Helliwell C.A., Stoutjesdijk P., Dennis E.S., Peacock W.J. (2001). Control of flowering time by *FLC* orthologues in *Brassica napus*. Plant J..

[16] Robert L.S., Robson F., Sharpe A., Lydiate D., Coupland G. (1998). Conserved structure and function of the *Arabidopsis* flowering time gene *CONSTANS* in *Brassica napus*. Plant Mol. Biol..

[17] Fadina O., Pankin A., Khavkin E. (2013). Molecular characterization of the flowering time gene *FRIGIDA* in *Brassica* genomes A and C. Russ. J. Plant Physl..

[18] Yi L., Chen C., Yin S., Li H., Li Z., Wang B., King G.J., Wang J., Liu K. (2018). Sequence variation and functional analysis of a *FRIGIDA* orthologue (*BnaA3.FRI*) in *Brassica napus*. BMC Plant Biol..

[19] Ying L., Chen H., Cai W. (2014). *BnNAC485* is involved in abiotic stress responses and flowering time in *Brassica napus*. Plant Physiol. Bioch..

[20] Xu J., Dai H. (2016). *Brassica napus Cycling Dof Factor1* (*BnCDF1*) is involved in flowering time and freezing tolerance. Plant Growth Regul..

[21] Schiessl S. (2020). Regulation and subfunctionalization of flowering time genes in the allotetraploid oil crop *Brassica napus*. Front. Plant Sci..

[22] Zhou E., Zhang Y., Wang H., Jia Z., Wang X., Wen J., Shen J., Fu T., Yi B. (2022). Identification and characterization of the MIKC-Type MADS-Box gene family in *Brassica napus* and its role in floral transition. Int. J. Mol. Sci..

[23] Treccarichi S., Di Gaetano C., Di Stefano F., Gasparini M., Branca F. (2021). Using simple sequence repeats in 9 *Brassica* complex species to assess hypertrophic curd induction. Agriculture.

[24] Treccarichi S., Di Gaetano C., Di Stefano F., Gasparini M., Branca F. (2023). Molecular markers for detecting inflorescence size of *Brassica oleracea* L. crops and *B. oleracea* complex species (n = 9) useful for breeding of broccoli (*B. oleracea* var. *italica*) and cauliflower (*B. oleracea* var. *botrytis*). Plants.

[25] Li H., Li J., Song J., Zhao B., Guo C., Wang B., Zhang Q., Wang J., King G.J., Liu K. (2019). An auxin signaling gene *BnaA3.IAA7* contributes to improved plant architecture and yield heterosis in rapeseed. New Phytol..

[26] Wang Y., Li J. (2008). Molecular basis of plant architecture. Annu. Rev. Plant Biol..

[27] Sun T. (2008). Gibberellin metabolism, perception and signaling pathways in *Arabidopsis*. Arab. Book.

[28] Sazuka T., Kamiya N., Nishimura T., Ohmae K., Sato Y., Imamura K., Nagato Y., Koshiba T., Nagamura Y., Ashikari M. (2009). A rice tryptophan deficient dwarf mutant, tdd1, contains a reduced level of indole acetic acid and develops abnormal flowers and organless embryos. Plant J..

[29] Jiang L., Liu X., Xiong G., Liu H., Chen F., Wang L., Meng X., Liu G., Yu H., Yuan Y. (2013). DWARF 53 acts as a repressor of strigolactone signalling in rice. Nature.

[30] Zhou F., Lin Q., Zhu L., Ren Y., Zhou K., Shabek N., Wu F., Mao H., Dong W., Gan L. (2013). D14-SCFD3-dependent degradation of D53 regulates strigolactone signalling. Nature.

[31] Zeng X., Zhu L., Chen Y., Qi L., Pu Y., Wen J., Yi B., Shen J., Ma C., Tu J. (2011). Identification, fine mapping and characterisation of a dwarf mutant (bnaC.dwf) in *Brassica napus*. Theor. Appl. Genet..

[32] Shen Y., Xiang Y., Xu E., Ge X., Li Z. (2018). Major co-localized QTL for plant height, branch initiation height, stem diameter, and flowering time in an alien introgression derived *Brassica napus* DH population. Front. Plant Sci..

[33] Wang Y., He J., Yang L., Wang Y., Chen W., Wan S., Chu P., Guan R. (2016). Fine mapping of a major locus controlling plant height using a high-density single-nucleotide polymorphism map in *Brassica napus*. Theor. Appl. Genet..

[34] Wang Y., Chen W., Chu P., Wan S., Yang M., Wang M., Guan R. (2016). Mapping a major QTL responsible for dwarf architecture in *Brassica napus* using a single-nucleotide polymorphism marker approach. BMC Plant Biol..

[35] Sun C., Wang B., Yan L., Hu K., Liu S., Zhou Y., Guan C., Zhang Z., Li J., Zhang J. (2016). Genome-wide association study provides insight into the genetic control of plant height in rapeseed (*Brassica napus* L.). Front. Plant Sci..

[36] Quijada P.A., Udall J.A., Lambert B., Osborn T.C. (2006). Quantitative trait analysis of seed yield and other complex traits in hybrid spring rapeseed (*Brassica napus* L.): 1. Identification of genomic regions from winter germplasm. Theor. Appl. Genet..

[37] Basunanda P., Radoev M., Ecke W., Friedt W., Becker H.C., Snowdon R.J. (2010). Comparative mapping of quantitative trait loci involved in heterosis for seedling and yield traits in oilseed rape (*Brassica napus* L.). Theor. Appl. Genet..

[38] Shi J., Li R., Qiu D., Jiang C., Long Y., Morgan C., Bancroft I., Zhao J., Meng J. (2009). Unraveling the complex trait of crop yield with quantitative trait loci mapping in *Brassica napus*. Genetics.

[39] Cai D., Xiao Y., Yang W., Ye W., Wang B., Younas M., Wu J., Liu K. (2014). Association mapping of six yield-related traits in rapeseed (*Brassica napus* L.). Theor. Appl. Genet..

[40] Zheng M., Peng C., Liu H., Tang M., Yang H., Li X., Liu J., Sun X., Wang X., Xu J. (2017). Genome-Wide association study reveals candidate genes for control of plant height, branch initiation height and branch number in rapeseed (*Brassica napus* L.). Front. Plant Sci..

[41] Li F., Chen B., Xu K., Gao G., Yan G., Qiao J., Li J., Li H., Li L., Xiao X. (2016). A genome-wide association study of plant height and primary branch number in rapeseed (*Brassica napus*). Plant Sci..

[42] Chen W., Zhang Y., Liu X.P., Chen B.Y., Tu J.X., Fu T. (2007). Detection of QTL for six yield-related traits in oilseed rape (*Brassica napus*) using DH and immortalized F2 populations. Theor. Appl. Genet..

[43] Zhao W., Wang X., Wang H., Tian J., Li B., Chen L., Chao H., Long Y., Xiang J., Gan J. (2016). Genome-Wide identification of QTL for seed yield and yield-related traits and construction of a high-density consensus map for QTL comparison in *Brassica napus*. Front. Plant Sci..

[44] Luo Z., Wang M., Long Y., Huang Y., Shi L., Zhang C., Liu X., Fitt B., Xiang J., Mason A.S. (2018). Correction to: Incorporating pleiotropic quantitative trait loci in dissection of complex traits: Seed yield in rapeseed as an example. Theor. Appl. Genet..

[45] Zheng M., Hu M., Yang H., Tang M., Zhang L., Liu H., Li X., Liu J., Sun X., Fan S. (2019). Three *BnaIAA7* homologs are involved in auxin/brassinosteroid-mediated plant morphogenesis in rapeseed (*Brassica napus* L.). Plant Cell Rep..

[46] Liu C., Wang J., Huang T., Wang F., Yuan F., Cheng X., Zhang Y., Shi S., Wu J., Liu K. (2010). A missense mutation in the VHYNP motif of a DELLA protein causes a semi-dwarf mutant phenotype in *Brassica napus*. Theor. Appl. Genet..

[47] Zhao B., Li H., Li J., Wang B., Dai C., Wang J., Liu K. (2017). *Brassica napus DS-3*, encoding a DELLA protein, negatively regulates stem elongation through gibberellin signaling pathway. Theor. Appl. Genet..

[48] Running M.P., Lavy M., Sternberg H., Galichet A., Gruissem W., Hake S., Ori N., Yalovsky S. (2004). Enlarged meristems and delayed growth in *plp* mutants result from lack of CaaX prenyltransferases. Proc. Natl. Acad. Sci. USA.

[49] Maurer-Stroh S., Washietl S., Eisenhaber F. (2003). Protein prenyltransferases. Genome Biol..

[50] McTaggart S.J. (2006). Isoprenylated proteins. Cell. Mol. Life Sci..

[51] Running M.P. (2014). The role of lipid post-translational modification in plant developmental processes. Front. Plant Sci..

[52] Hemsley P.A. (2014). The importance of lipid modified proteins in plants. New Phytol..

[53] Galichet A., Gruissem W. (2003). Protein farnesylation in plants—Conserved mechanisms but different targets. Curr. Opin. Plant Biol..

[54] Wang Y., Beaith M., Chalifoux M., Ying J., Uchacz T., Sarvas C., Griffiths R., Kuzma M., Wan J., Huang Y. (2009). Shoot-specific down-regulation of protein farnesyltransferase (α-subunit) for yield protection against drought in canola. Mol. Plant.

[55] Yang Z., Liang C., Wei L., Wang S., Yin F., Liu D., Guo L., Zhou Y., Yang Q. (2022). BnVIR: Bridging the genotype-phenotype gap to accelerate mining of candidate variations underlying agronomic traits in *Brassica napus*. Mol. Plant.

[56] Yu K., Wang X., Chen F., Chen S., Peng Q., Li H., Zhang W., Hu M., Chu P., Zhang J. (2016). Genome-wide transcriptomic analysis uncovers the molecular basis underlying early flowering and apetalous characteristic in *Brassica napus* L.. Sci. Rep..

[57] Yu K., Wang X., Li W., Sun L., Peng Q., Chen F., Zhang W., Guan R., Zhang J. (2019). Identification and physical mapping of QTLs associated with flowering time in *Brassica napus* L.. Euphytica.

[58] Huang L., Min Y., Schiessl S., Xiong X., Jan H.U., He X., Qian W., Guan C., Snowdon R.J., Hua W. (2021). Integrative analysis of GWAS and transcriptome to reveal novel loci regulation flowering time in semi-winter rapeseed. Plant Sci..

[59] Wickramasinghe H., Miura H. (2003). Gene dosage effect of the wheat *Wx* alleles and their interaction on amylose synthesis in the endosperm. Euphytica.

[60] Wang X., Yu K., Li H., Peng Q., Chen F., Zhang W., Chen S., Hu M., Zhang J. (2015). High-density SNP map construction and QTL identification for the apetalous character in *Brassica napus* L.. Front. Plant Sci..

[61] Song J.M., Guan Z., Hu J., Guo C., Yang Z., Wang S., Liu D., Wang B., Lu S., Zhou R. (2020). Eight high-quality genomes reveal pan-genome architecture and ecotype differentiation of *Brassica napus*. Nat. Plants.

[62] Wang X., Wang H., Wang J., Sun R., Wu J., Liu S., Bai Y., Mun J.H., Bancroft I., Cheng F. (2011). The genome of the mesopolyploid crop species *Brassica rapa*. Nat. Genet..

[63] Liu S., Liu Y., Yang X., Tong C., Edwards D., Parkin I.A., Zhao M., Ma J., Yu J., Huang S. (2014). The *Brassica oleracea* genome reveals the asymmetrical evolution of polyploid genomes. Nat. Commun..

[64] Tamura K., Dudley J., Nei M., Kumar S. (2007). MEGA4: Molecular Evolutionary Genetics Analysis (MEGA) software version 4.0. Mol. Biol. Evol..

[65] Zhang K., He J., Liu L., Xie R., Qiu L., Li X., Yuan W., Chen K., Yin Y., Kyaw M. (2020). A convenient, rapid and efficient method for establishing transgenic lines of *Brassica napus*. Plant Methods.

[66] Clough S.J., Bent A.F. (1998). Floral dip: A simplified method for *Agrobacterium*-mediated transformation of *Arabidopsis thaliana*. Plant J..

[67] Yang M., Yang Q., Fu T., Zhou Y. (2011). Overexpression of the *Brassica napus BnLAS* gene in *Arabidopsis* affects plant development and increases drought tolerance. Plant Cell Rep..

[68] Broun P., Poindexter P., Osborne E., Jiang C.Z., Riechmann J.L. (2004). *WIN1*, a transcriptional activator of epidermal wax accumulation in Arabidopsis. Proc. Natl. Acad. Sci. USA.

[69] Livak K., Schmittgen T. (2001). Analysis of relative gene expression data using real-time quantitative PCR and the 2(-Delta Delta C(T)) Method. Methods.

[70] Wang X., Wang H., Long Y., Liu L., Zhao Y., Tian J., Zhao W., Li B., Chen L., Chao H. (2015). Dynamic and comparative QTL analysis for plant height in different developmental stages of *Brassica napus* L.. Theor. Appl. Genet..

[71] Yu K., Zhang W., Guo Y., Zheng M., Chen F., Sun C., Hu M., Tian E., Wang X., Zhang J. (2021). Integrating unconditional and conditional QTLs to dissect the genetic basis of stem mechanical strength in *Brassica napus* L.. Euphytica.

