# Supplementary material for: BnPLP1 Positively Regulates Flowering Time, Plant Height, and Main Inflorescence Length in Brassica napus
Source: Genes (Basel). 2023 Dec 13;14(12):2206. doi: 10.3390/genes14122206 (PMC10743044; doi:10.3390/genes14122206)
Supplement: Supplementary file 1 [file genes-14-02206-s001.zip › Supplementary materials/Supplementary File 1.pdf]

Sequence Identity: 72.6%

```
BnPLP1_DNA ---------------------------------------------------------TAATCTGTCTTCGCATCCCCTTA 23
BnPLP2_DNA ACACTGAGATCTAGTTCTTGAGGTCTGTGTCCTTGTCCAATTACTTTGTCTTTCTTCACATCCTCAAACCCACCCTAATA 80

BnPLP1_DNA AACCCTAGTTTGAATCTGTTTGATTTTGGGCATCAGAGTAG----------------------GTTTTCGATCAGTCGTG 81
BnPLP2_DNA CTCCTCAAATTATCACTCTCTTAGATTAGGGATCCAAGTCGGAACCCTAATTTGAATCGTCTTGATTTCGATCAGTTGTG 160

BnPLP1_DNA ---------AGGAAACATGGATTCCGACGCGACCGTGCCACTGAGCGAGCGCCCGGAGTGGTCCGACGTTGTCCCGTTGA 152
BnPLP2_DNA TGCAAAGGGAGGAAACATGGATTCCGACTCAACCGTGCCACTGAGCGAGCGCCCGGAGTGGTCTGACGTTGTCCCGTTGA 240

BnPLP1_DNA GTCAGGACGATGGTCCGAACCCGGTGGTTCCGATCGCTTACAAGGAAGACTTCCGGGAGACGATGGATTACTTCCGGGCG 232
BnPLP2_DNA CTCAGGACGATGGTCCGAACCCGGTGGTTCGGATCGCTTACAAGGAAGACTTCCGCGAGACGATGGATTACTTCCGGGCG 320

BnPLP1_DNA ATTTACCGCTCCGACGAGCGTTCTCCTCGCGCGCTGCGACTCACGGAAGAAGCTCTCCGCTTAAACTCCGGCAACTACAC 312
BnPLP2_DNA ATTTACCGCTCCGACGAGCGTTCTGCTCGCGCGCTGCGACTCACGGAAGAAGCTCTCCGCTTAAACTCGGGCAACTACAC 400

BnPLP1_DNA CGTAACTGATCCTTCTTCTTTG-CCCTACTACTACTCTTTGATTTGATTAGATTGTAACTCTTCTTGTTCTTGTTGTTCC 391
BnPLP2_DNA CGTAACTGATCCTTCTTCTTTTGCCCTAGTACTACTGTTCTTAAAGAGTTGGAT----------TTGATTAGATTGTAAC 470

BnPLP1_DNA TTTTTGAAGGTGTGGCACTTCAGGCGCTTAGTACTGGAGGAGCTTGATCACGACTTGTATGAAGAGCTCAAGTTCATCGA 471
BnPLP2_DNA TTTTTGAAGGTGTGGCACTTCAGGCGCTTAGTACTCGAGGAGCTTAATAACGACTTGTATGAAGAGCTCAAGTTCATCGA 550

BnPLP1_DNA AAGCATTGCTGAGGATAACTCCAAGAACTACCAGTTGTGGTAAAAAGTTCATTCCTTTTTGATTTTTTTCATTACACGTT 551
BnPLP2_DNA AAGCATTGCTGAGGATAACTCTAAGAACTACCAGTTGTGGTAAAAAGTTCATACCTTTTTTGTCTCTTTGGTTTGTTCAT 630

BnPLP1_DNA TGTTTATACTGTGAAACAACAACTGTATACGTCTACTATATGCTTTCGTGGGAATCATTTAACTTTTATATGTGAGATGA 631
BnPLP2_DNA G-----------------------------------AAAGTTCTTTCAATAGTGTCTTTAGAATTTTAAACATGTGTT-- 673

BnPLP1_DNA AAGTTTGCTCTTTTTAAAGTCCATAGTGTTAAGCATATCTGCTGCTTTAAGAAAGTTCCTACTCTTTGTTTCTTTTTTTC 711
BnPLP2_DNA -----------------------------------------------------------------------CCTTTGTTC 682

BnPLP1_DNA ATGCGATGAGTAGTGTTTGTATATATACTGTGAAATTGACTGATGAATAGTTTTGAACCAAGAACTGTATATGTTTTGCA 791
BnPLP2_DNA ATGCTTCTCTAGATTTAGATGAAAATTCTTTAGTGTTAGCTTATCTGATGCTTTAAGTAAG------------------- 743

BnPLP1_DNA TTTTTGGAATTCATTAGAACCTGAAAGACGTGTTACATAGTGCATCTTCTGTATATTTGAGATGAAAGCTTGCTCATCTG 871
BnPLP2_DNA -------------------------------------------------------------------------------- 743

BnPLP1_DNA CTGCTTTATGGAAGTTTAGCTTTGTCTGCTGGCTTTGATTTGTGATGCTCAATGGCTCTGCTCTAATTTGTTTTTCATTT 951
BnPLP2_DNA -----------TTAACAACCTTCGTTTGTTTGCTTTGATTTTTGATGATCAATGGTTCTGCTCTAACTT--TTTTCCTTT 810

BnPLP1_DNA TGTTCTAGGCATCATCGGAGATGGGTTGCAGAGAAACTGGGTCCTGATGTTGCAGGAAAGGAACTTGACTTTACTCGGAG 1031
BnPLP2_DNA TGTTCTAGGCATCATCGACGATGGGTTGCAGAGAAACTGGGTCCTGATGTTGCAGGAAAGGAACTTGAGTTTACTCGGAG 890

BnPLP1_DNA CATACTATCAGTTGATGCCAAACATTATCATGCTTGGTCACATAGGCAGGTTTGTCATCTCGAAACATTGCTTAACCCAA 1111
BnPLP2_DNA GGTACTATCACTTGATGCCAAGCATTATCATGCTTGGTCACATAGGCAGGTTTGTCATCTCAAAACATAATATAACTCAC 970

BnPLP1_DNA TCACTTGACGTG-----TAGTATTATCTTTTTGCTTATTGTTGGTAAACAATCTACATTATTATGCTTAGTCACATAGGC 1186
BnPLP2_DNA TCACTTGATGATGTTTATAGTATTATCTTTTTGCTTGAT-TTGGGAAACAATGTACATTATCATGCTTAATCACATAGGC 1049

BnPLP1_DNA AGGTTTTTTAATCTCAGAACCTTGTTTAAAGCACTCACTATGTATGCTATTTTTTTGCTTTATGTTGGGAAACAATCTG 1266
BnPLP2_DNA AGGTTTTTTAATCTCAGAACCTTGTTTAAAGAACTTACTATGGATGTTATCATCTTTGCTTGATGTTGGGAAACAATCTG 1129

BnPLP1_DNA TAGTGGGCACTACAAGCATTAGGTGGATGGGAAAATGAGCTTGATTACTGTCATGAGCTCCTTGAAGCTGACGTCTTTAA 1346
BnPLP2_DNA CAGTGGGCGCTACAAGCATTAGGAGGATGGGAAAATGAGCTTAACTACTGCCACGAGCTCCTTGAAGCTGACGTCTTTAA 1209

BnPLP1_DNA CAACTCTGCATGGAATCAGGTAAATATGATTCATCAATACACAATGCTAGATTATGTTTCCCAAATTATAATTG------ 1420
BnPLP2_DNA CAACTCTGCATGGAATCAGGTAAATATGATTCATCAATACACATG-CTAAATTATGTTTCCCAAATTATCATTGGCAATT 1288

BnPLP1_DNA -CCTTCTTAAATCAAATCTTGCAGAGGTATTACGTTATAACTAGATCACCTTCGTTGGGGGGCCTAAAACCCATGAGAGA 1499
BnPLP2_DNA CAGTCTTCTTTTTAAATCTTGCAGAGGTATTACGTTATAACTAGATCACCTTCGTTGGGAGGCCTAGAAGCCATGAGAGA 1368

BnPLP1_DNA ATCTGAAGTAAGCTACACAGTCAAAGCCATTTTAGCAAATCCCGGGAACGAGAGCTCTTGGAGATACCTGAAAGCCCTTT 1579
BnPLP2_DNA ATCTGAAGTAAGCTACACAGTCAAAGCCATTTTAGCAAATCCCGGGAACGAGAGCTCTTGGAGGTACCTGAAAGCCCTTT 1448

BnPLP1_DNA ACAAAGACGACACAGAGTCTTGGATTAGTGATCCAAGTGTTTCCTCAGTCTGTTTGAAAGTTCTGTCACGCACGGACTGC 1659
BnPLP2_DNA ACAAAGACGACACAGAGTCTTGGATTAGTGATCCAAGTGTTTCCTCAGTCTGTTTGAAAGTTCTCTCACGCACGGACTGC 1528

BnPLP1_DNA TTCCATGGATTCGCTCTGAGCACCCTTTTGGATCTTCTGTGCGATGGGTTGAGACCAACCAACGAGCATATAGACTCGGT 1739
BnPLP2_DNA TTCCATGGATTCGCTCTGAGCACCCTTTTGGATCTTCTGTGCGATGGGTTGAGACCAACCAACGAGCATAGAGACTCGGT 1608

BnPLP1_DNA GAAAGCTCTAGCTAATGAAGATCCAGAGGCTAACTTGGCCAATTTGGTGTGTACCATTCTGTGTCGTGTTGATCCTATAA 1819
BnPLP2_DNA GAAAGCTCTAGCTAATGAAGAACCAGAGACTAACTTGGCCAATTTGGTGTGTACCATTCTGTGTCGTGTTGATCCTATAA 1688

BnPLP1_DNA GAGCTAACTATTGGGCATGGAGGAAGAGCAAAATTACAGTGGCAATATAACCACACTTGAAAAAGTTATTTACTTTTACG 1899
BnPLP2_DNA GAGCTAACTATTGGGCATGGAGGAAGAGCAAGATTACAGTGGCAATATAACCACACTTGAGAAAGTTATTTATTTTTACG 1768
```

```
BnPLP1_DNA TTTTAAACTACTGCCTTTTTCATGAAACATGTTACCATACATCACAACAAATCTTGATTTTCTTGGCTTCCCTTTTTATT 1979
BnPLP2_DNA TTTTAATCTACTGCTTTTTTCATGAAACATG-----ATGCATCAAAACAAATCTCGATTTCTCGG------------CTT 1831

BnPLP1_DNA TTATTTTTATCAATGCAAAATCTAATGAATGTCAACACTAACGTGTAGTATTGGGTTTGGAGTATTCAAACCCGGTTTCA 2059
BnPLP2_DNA CCCTTTTTATCAATGCAAAATCTAATGACTAACCTGCAGTATTG--------GGTTTTGGAAAATTCGAACCCGGTTTCA 1903

BnPLP1_DNA TCAAATAAAAAATACAATCCCGGCTTGGGATAGATTCCGGTTCAACATTATGATTGTTGGACATGAAGGGAGAATGTGGT 2139
BnPLP2_DNA GCATATAAAAACATCCAATCGCCGTTTGGGATGGATTCCGGGTCAAAATATGATTGTTGGACATGAAGGGAGAATGTGGT 1983

BnPLP1_DNA CACGTAA------------------------------------------------------------------ 2146
BnPLP2_DNA CACGTAATTGCTTTGTCTTTTCTGTGTGTTGTTGTATATGACAGAATGTGTGGTGTTTGTGATATGTGAATGG 2056
```
