# Supplementary material for: BnPLP1 Positively Regulates Flowering Time, Plant Height, and Main Inflorescence Length in Brassica napus
Source: Genes (Basel). 2023 Dec 13;14(12):2206. doi: 10.3390/genes14122206 (PMC10743044; doi:10.3390/genes14122206)
Supplement: Supplementary file 1 [file genes-14-02206-s001.zip › Supplementary materials/Supplementary File 2.pdf]

Sequence Identity: 96.5%

```
BnPLP1_CDS ATGGATTCCGACGCGACCGTGCCACTGAGCGAGCGCCCGGAGTGGTCCGACGTTGTCCCGTTGAGTCAGGACGATGGTCC 80
BnPLP2_CDS ATGGATTCCGACTCAACCGTGCCACTGAGCGAGCGCCCGGAGTGGTCTGACGTTGTCCCGTTGACTCAGGACGATGGTCC 80

BnPLP1_CDS GAACCCGGTGGTTCCGATCGCTTACAAGGAAGACTTCCGGGAGACGATGGATTACTTCCGGGCGATTTACCGCTCCGACG 160
BnPLP2_CDS GAACCCGGTGGTTCGGATCGCTTACAAGGAAGACTTCCGCGAGACGATGGATTACTTCCGGGCGATTTACCGCTCCGACG 160

BnPLP1_CDS AGCGTTCTCCTCGCGCGCTGCGACTCACGGAAGAAGCTCTCCGCTTAAACTCCGGCAACTACACCGTGTGGCACTTCAGG 240
BnPLP2_CDS AGCGTTCTGCTCGCGCGCTGCGACTCACGGAAGAAGCTCTCCGCTTAAACTCGGGCAACTACACCGTGTGGCACTTCAGG 240

BnPLP1_CDS CGCTTAGTACTGGAGGAGCTTGATCACGACTTGTATGAAGAGCTCAAGTTCATCGAAAGCATTGCTGAGGATAACTCCAA 320
BnPLP2_CDS CGCTTAGTACTCGAGGAGCTTAATAACGACTTGTATGAAGAGCTCAAGTTCATCGAAAGCATTGCTGAGGATAACTCTAA 320

BnPLP1_CDS GAACTACCAGTTGTGGCATCATCGGAGATGGGTTGCAGAGAAACTGGGTCCTGATGTTGCAGGAAAGGAACTTGACTTTA 400
BnPLP2_CDS GAACTACCAGTTGTGGCATCATCGACGATGGGTTGCAGAGAAACTGGGTCCTGATGTTGCAGGAAAGGAACTTGAGTTTA 400

BnPLP1_CDS CTCGGAGCATACTATCAGTTGATGCCAAACATTATCATGCTTGGTCACATAGGCAGTGGGCACTACAAGCATTAGGTGGA 480
BnPLP2_CDS CTCGGAGGGTACTATCACTTGATGCCAAGCATTATCATGCTTGGTCACATAGGCAGTGGGCGCTACAAGCATTAGGAGGA 480

BnPLP1_CDS TGGGAAAATGAGCTTGATTACTGTCATGAGCTCCTTGAAGCTGACGTCTTTAACAACTCTGCATGGAATCAGAGGTATTA 560
BnPLP2_CDS TGGGAAAATGAGCTTAACTACTGCCACGAGCTCCTTGAAGCTGACGTCTTTAACAACTCTGCATGGAATCAGAGGTATTA 560

BnPLP1_CDS CGTTATAACTAGATCACCTTCGTTGGGGGGCCTAAAACCCATGAGAGAATCTGAAGTAAGCTACACAGTCAAAGCCATTT 640
BnPLP2_CDS CGTTATAACTAGATCACCTTCGTTGGGAGGCCTAGAAGCCATGAGAGAATCTGAAGTAAGCTACACAGTCAAAGCCATTT 640

BnPLP1_CDS TAGCAAATCCCGGGAACGAGAGCTCTTGGAGATACCTGAAAGCCCTTTACAAAGACGACACAGAGTCTTGGATTAGTGAT 720
BnPLP2_CDS TAGCAAATCCCGGGAACGAGAGCTCTTGGAGGTACCTGAAAGCCCTTTACAAAGACGACACAGAGTCTTGGATTAGTGAT 720

BnPLP1_CDS CCAAGTGTTTCCTCAGTCTGTTTGAAAGTTCTGTCACGCACGGACTGCTTCCATGGATTCGCTCTGAGCACCCTTTTGGA 800
BnPLP2_CDS CCAAGTGTTTCCTCAGTCTGTTTGAAAGTTCTCTCACGCACGGACTGCTTCCATGGATTCGCTCTGAGCACCCTTTTGGA 800

BnPLP1_CDS TCTTCTGTGCGATGGGTTGAGACCAACCAACGAGCATATAGACTCGGTGAAAGCTCTAGCTAATGAAGATCCAGAGGCTA 880
BnPLP2_CDS TCTTCTGTGCGATGGGTTGAGACCAACCAACGAGCATAGAGACTCGGTGAAAGCTCTAGCTAATGAAGAACCAGAGACTA 880

BnPLP1_CDS ACTTGGCCAATTTGGTGTGTACCATTCTGTGTCGTGTTGATCCTATAAGAGCTAACTATTGGGCATGGAGGAAGAGCAAA 960
BnPLP2_CDS ACTTGGCCAATTTGGTGTGTACCATTCTGTGTCGTGTTGATCCTATAAGAGCTAACTATTGGGCATGGAGGAAGAGCAAG 960

BnPLP1_CDS ATTACAGTGGCAATATAA 978
BnPLP2_CDS ATTACAGTGGCAATATAA 978
```
