# Supplementary figures and images for: BnPLP1 Positively Regulates Flowering Time, Plant Height, and Main Inflorescence Length in Brassica napus
Source: Genes (Basel). 2023 Dec 13;14(12):2206. doi: 10.3390/genes14122206 (PMC10743044; doi:10.3390/genes14122206)

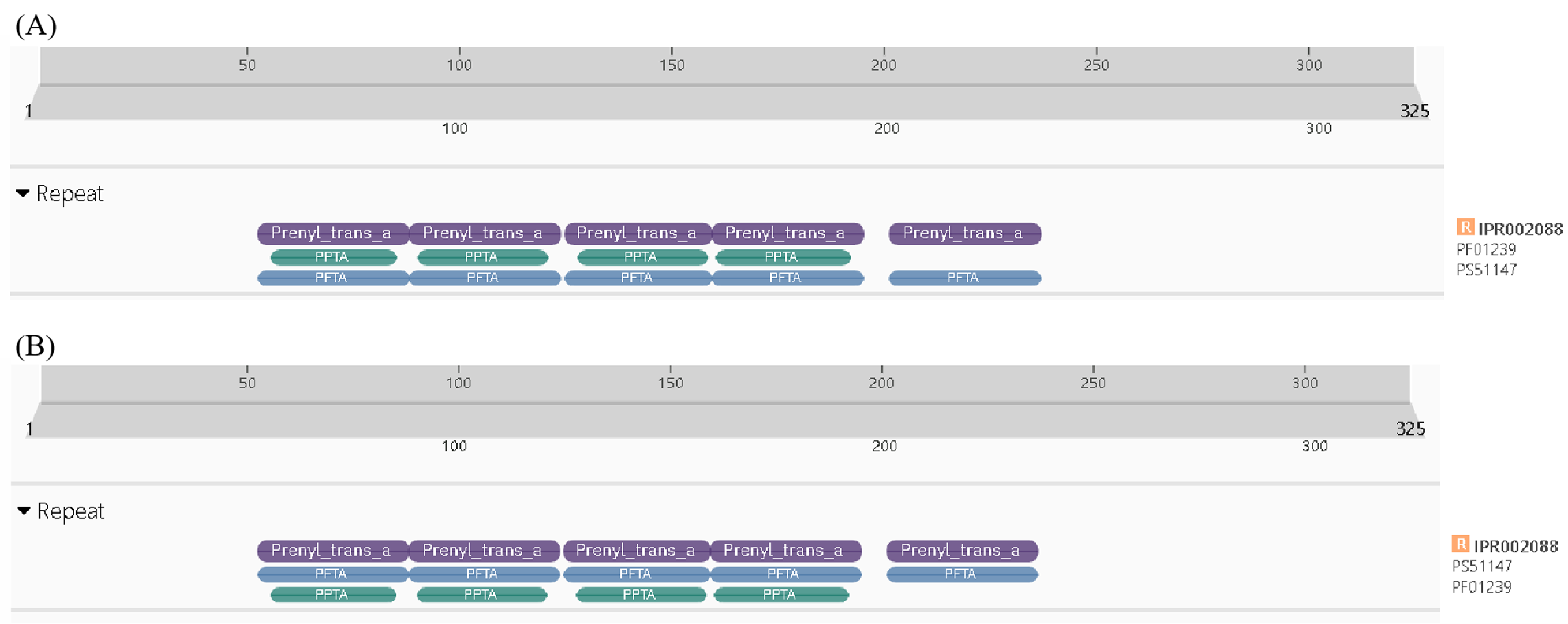

Supplement: Supplementary file 1 [file genes-14-02206-s001.zip › Supplementary materials/Figure S1.tif]

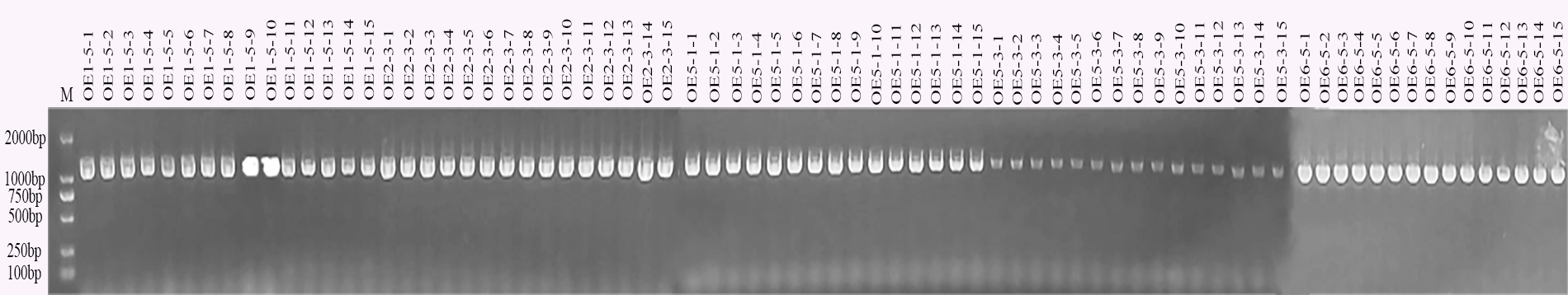

Supplement: Supplementary file 1 [file genes-14-02206-s001.zip › Supplementary materials/Figure S2.tif]

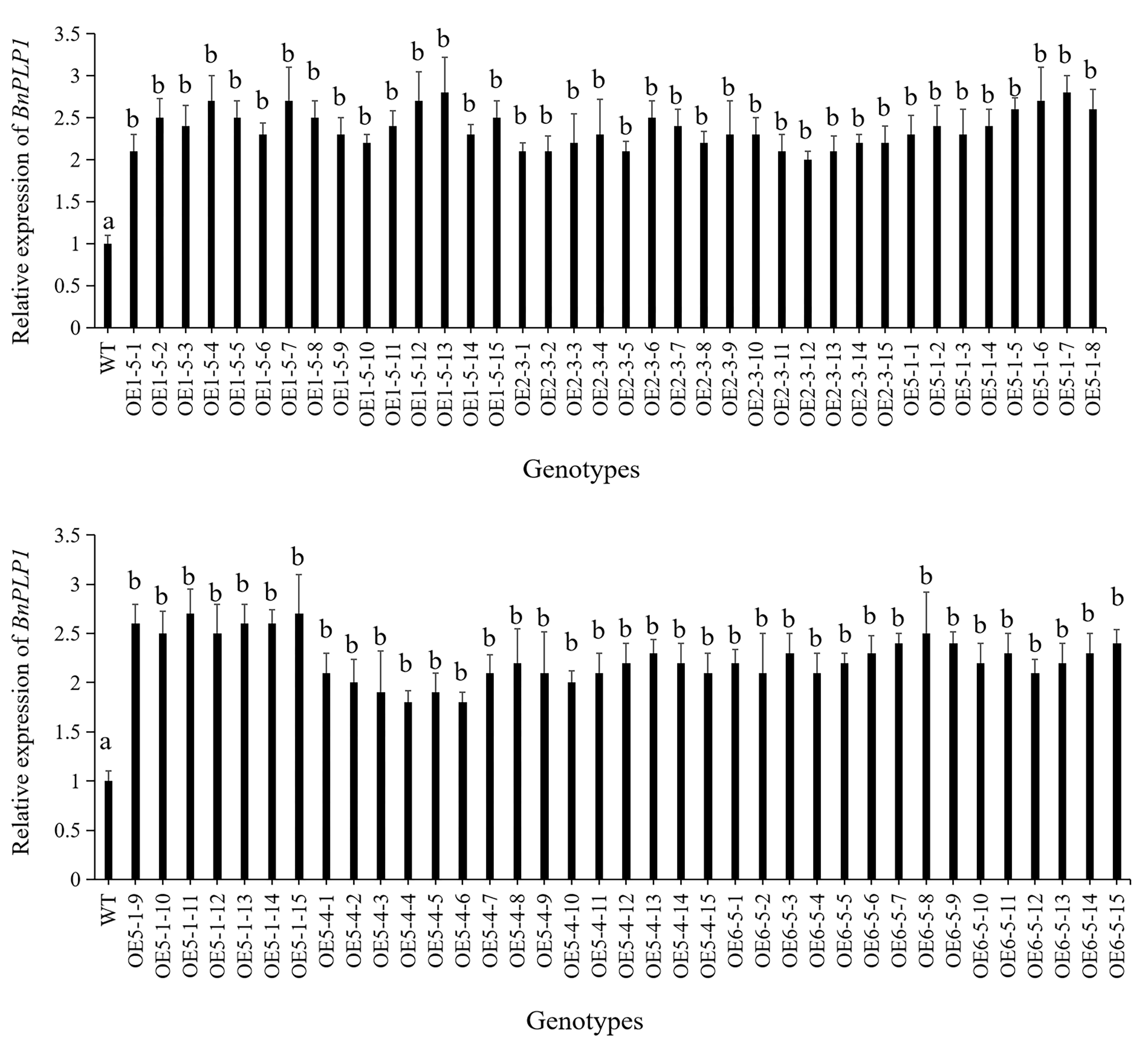

Supplement: Supplementary file 1 [file genes-14-02206-s001.zip › Supplementary materials/Figure S3.tif]

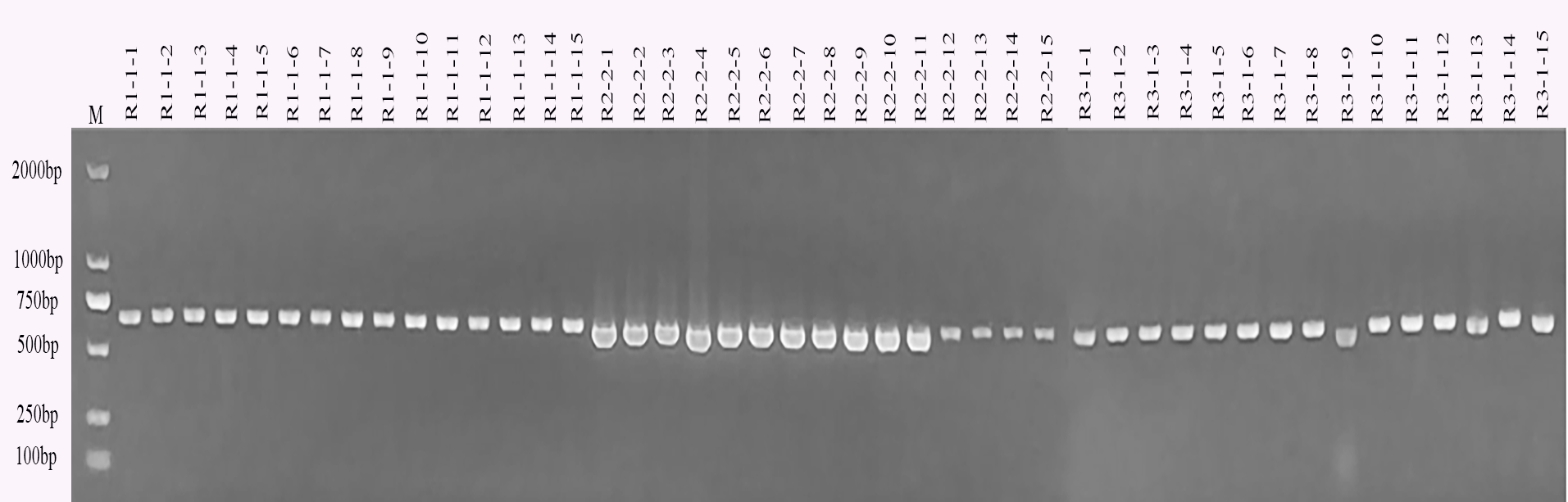

Supplement: Supplementary file 1 [file genes-14-02206-s001.zip › Supplementary materials/Figure S4.tif]

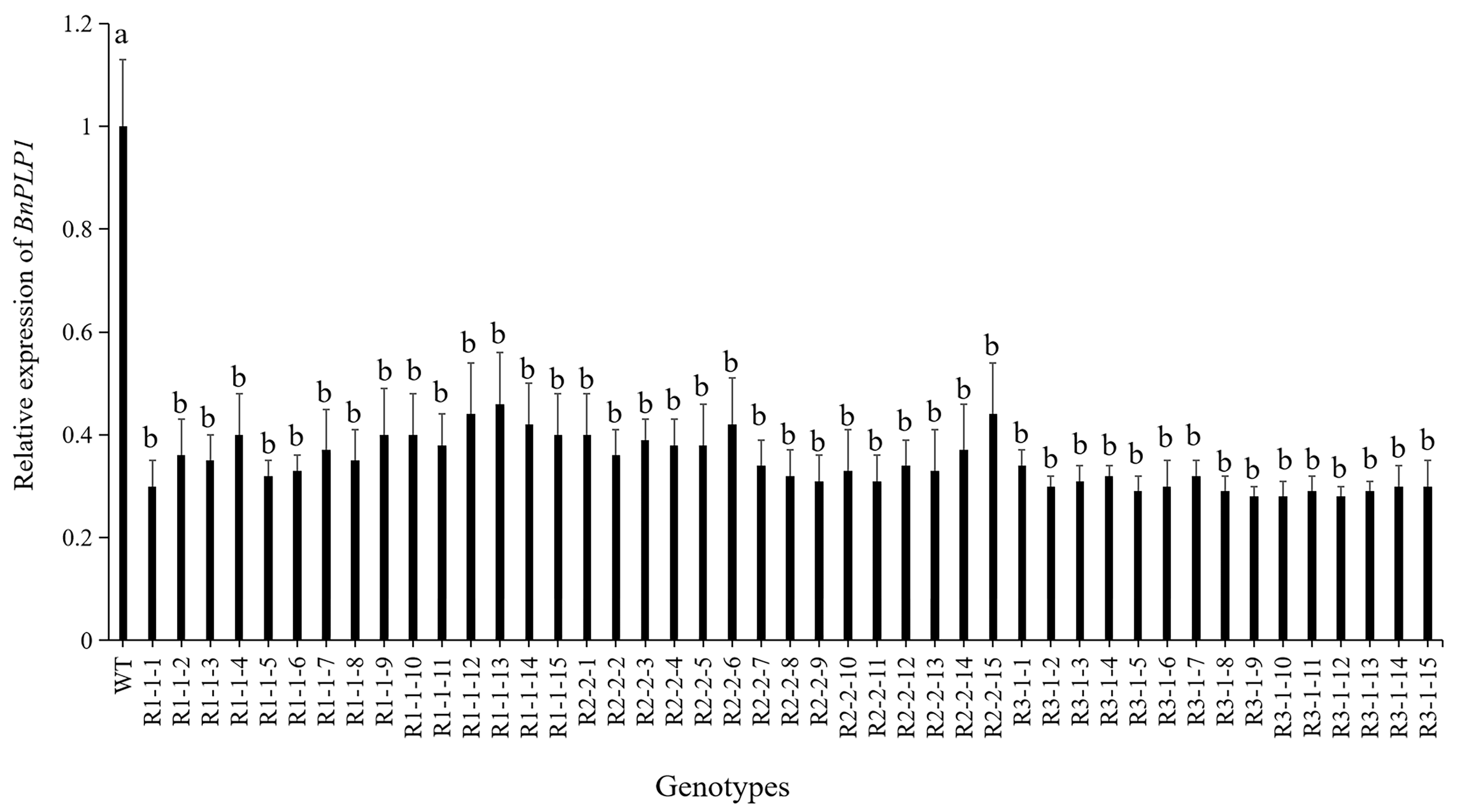

Supplement: Supplementary file 1 [file genes-14-02206-s001.zip › Supplementary materials/Figure S5.tif]

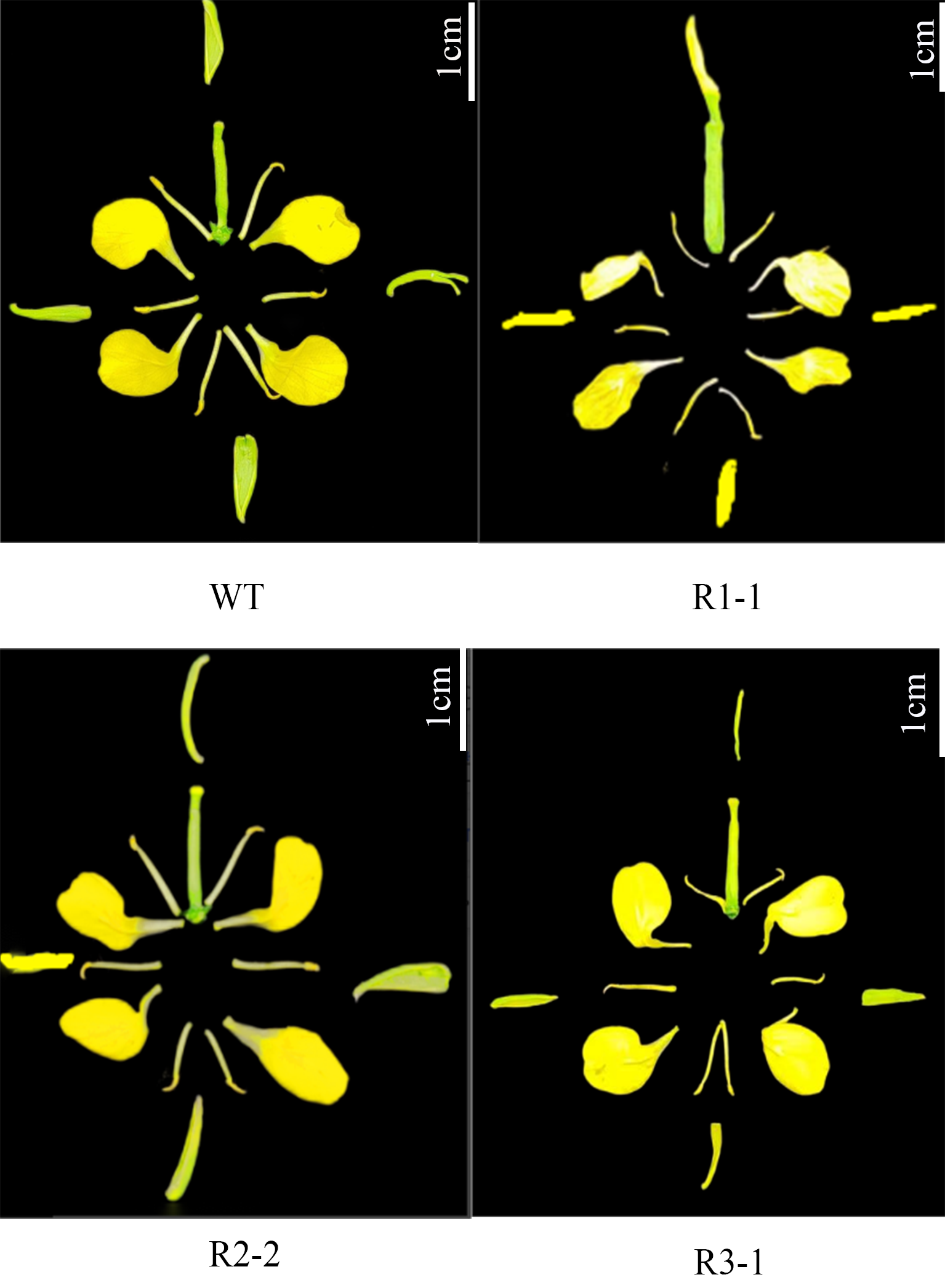

Supplement: Supplementary file 1 [file genes-14-02206-s001.zip › Supplementary materials/Figure S6.tif]
